# Downregulation of CLDN7 due to promoter hypermethylation is associated with human clear cell renal cell carcinoma progression and poor prognosis

**DOI:** 10.1186/s13046-018-0924-y

**Published:** 2018-11-14

**Authors:** Yifan Li, Yanqing Gong, Xianghui Ning, Ding Peng, Libo Liu, Shiming He, Kan Gong, Cuijian Zhang, Xuesong Li, Liqun Zhou

**Affiliations:** 1 Department of Urology, Peking University First Hospital, Beijing, 100034 China; 2 Institute of Urology, Peking University, Beijing, 100034 China; 3 National Urological Cancer Center, Beijing, 100034 China; 4 Department of Urology, The First Affiliated Hospital of Zhengzhou University, Henan, 450052 China

**Keywords:** ccRCC, CLDN7, Methylation, Tumor suppressor, Apoptosis, EMT

## Abstract

**Background:**

Metastasis is the primary cause of death in renal cell carcinoma (RCC). Loss of cell-to-cell adhesion, including tight junctions (TJs) is the initial step in the process of metastasis. Claudin-7 (CLDN7) is a major component of TJs. However, the clinical significance and its regulation of kidney tumorigenesis remain poorly understood.

**Methods:**

A total of 120 fresh clear cell RCC (ccRCC) specimens and 144 primary RCC and adjacent nonmalignant renal paraffin specimens were obtained from Department of Urology, Peking University First Hospital. Expression of CLDN7 in ccRCC tissues and cell lines were determined using bioinformatic data mining, quantitative real-time PCR (qRT-PCR), Western blotting and immunostaining. The clinical significance of CLDN7 expression and promoter DNA methylation status was analyzed in ccRCC patients from Peking University First Hospital and The Cancer Genome Atlas. Additionally, the methylation specific-PCR, bisulfite genomic sequencing and demethylation analysis of CLDN7 were performed. Biological functions of CLDN7 were investigated by examining cell proliferation using MTS assays and EdU incorporation assays, cell migration by in vitro wound healing assays and transwell migration assays, cell invasion by transwell invasion assays, and cell apoptosis by flow cytometry. Mouse model experiments were performed to confirm the effects of CLDN7 on tumor growth and metastasis in vivo. The molecular mechanism of CLDN7 function was investigated using gene-set enrichment analysis (GSEA) and high-throughput cDNA sequencing (RNA-Seq) and confirmed by qRT-PCR, Western blot and immunostaining in vitro and in vivo.

**Results:**

Our findings revealed that CLDN7 is frequently downregulated via hypermethylation of its promoter in ccRCC. CLDN7 can help predict aggressive tumor status and poor prognosis in ccRCC patients. Interestingly, hypermethylation of the CLDN7 promoter was related to advanced ccRCC status and poor prognosis. Moreover, overexpression of CLDN7 induced cell apoptosis, suppressed proliferation, migration and invasion abilities of ccRCC cells both in vitro and in vivo. Additionally, GSEA and RNA-Seq results showed that CLDN7 had negative effects in cancer-associated signaling pathways and (epithelial-mesenchymal transition) EMT-related pathways. These results were validated by qRT-PCR, Western blot and immunostaining.

**Conclusions:**

We have demonstrated a previously undescribed role of CLDN7 as a ccRCC suppressor and suggest that loss of CLDN7 potentiates EMT and tumor progression. CLDN7 may serve as a functional tumor suppressor in tumor progression and a potential biomarker and target in patients with ccRCC.

**Electronic supplementary material:**

The online version of this article (10.1186/s13046-018-0924-y) contains supplementary material, which is available to authorized users.

## Background

Renal cell carcinoma (RCC) accounts for up to 85% to 90% of all kidney cancers [1, 2]. According to the most recent pathological classification by the International Society of Urological Pathology (ISUP), RCC mainly includes clear cell (ccRCC), papillary (pRCC) and chromophobe (chRCC) subtypes [3], with ccRCC being the most common subtype [4]. The 5-year survival rate of patients with kidney cancer increased approximately 30% in recent years. This improvement is largely due to low-stage and low-grade tumors being incidentally detected by improved early-detection techniques [2, 5, 6]. However, one third of patients with kidney cancer present with metastatic disease [5], and 20%~ 30% of the patients who undergo curative surgery relapse in distant sites during follow-up [7]. Although there has been considerable progress in the systemic treatment of metastatic renal cell carcinoma in the past 20 years [2], metastatic RCC remains an incurable condition for the majority of patients.

Metastasis is the primary cause of death for RCC. The initial step in the process of metastasis is the loss of cell-to-cell adhesion in the neoplastic epithelium [8]. Tight junctions (TJs) are the most apical components of the epithelial cell junctional complex and provide a form of cell–cell adhesion [9]. Claudin-7 (CLDN7) is a major component of TJs in epithelial cells. In RCC, CLDN7 is a marker of renal tumors originating from the distal nephron marker, such as chRCC and oncocytoma [10]. The expression of CLDN7 is significantly lower in ccRCC than non-ccRCC [10–12]. However, the clinical significance and molecular mechanisms of downregulation of CLDN7 in ccRCC remain unknown.

In this study, we confirmed that downregulation of CLDN7 due to hypermethylation may help predict aggressive tumor status and poor prognosis in ccRCC patients. Using combined data from human patients and in vitro and in vivo analyses in ccRCC cells manipulated for CLDN7 overexpression, we previously demonstrated a tumor suppressing role of CLDN7 and that it induced epithelial features in ccRCC cells. Bioinformatics and RNA sequencing analysis showed that genes that were influenced by CLDN7 overexpression were mostly enriched in cancer- and EMT-related pathways, indicating that CLDN7 may have an important role in the development and progression of ccRCC.

## Methods

### Human tissues

For DNA and RNA extraction, a total of 120 patients who had been pathologically diagnosed with ccRCC were included, and detailed clinicopathological features of the samples are summarized in Additional file 1: Table S1. A total of 120 paired ccRCC tissues and adjacent normal kidney tissues were immediately snap-frozen in liquid nitrogen following surgical resection. For immunohistochemical (IHC) staining, 144 patients were selected who had RCC and had undergone radical or partial nephrectomy between May 1, 2012 and August 30, 2012 at Peking University First Hospital. All the RCC paraffin specimens were pathologically diagnosed as RCC, including 129 ccRCC, 7 chRCC, 7 pRCC and 1 collecting duct RCC. This study was approved by ethics committee of the Peking University First Hospital (Beijing, China). Written informed consent was also obtained from all patients.

### Bioinformatic data mining

We downloaded TCGA RCC RNA-Seq gene expression data, TCGA ccRCC Methylation 450 K data and clinical data from UCSC Xena (http://xena.ucsc.edu/). A total of 129 normal kidney, 534 ccRCC, 66 chRCC and 291 pRCC tissues had CLDN7 mRNA expression, and 319 ccRCC tissues had CLDN7 promoter methylation data. All 534 ccRCC tumors had clinical data for clinical correlation and survival analyses. The heat map and the correlation between CLDN7 expression and methylation status were further analyzed in the same patient cohort and verified using UCSC Xena data. The relationship between CLDN7 mRNA expression and promoter methylation status was searched in the ccRCC cohort in TCGA database using cBioportal (http://cbioportal.org). Additionally, gene-set enrichment analysis (GSEA) was performed to compare differences in molecular pathways in cell processes between the low CLDN7 and high CLDN7 groups on the data from the ccRCC dataset of TCGA.

### Cell culture and transfection

The cell lines (HEK-293, Caki-2, Caki-1, OS-RC-2, 786-O, 769-P, ACHN and A498) were obtained from the American Type Culture Collection (Rockville, MD, USA). Cell lines were cultured according to conditions specified by the provider. The kidney cancer cell line KETR-3 was purchased from KeyGEN BioTECH Co., Ltd. (Jiangsu, China). For overexpression of CLDN7, recombinant pGC-LV-GV287-GFP vectors with the CLDN7 mRNA (NM_001185022.1) or with a scrambled control sequence (Control) were constructed by the Genechem Company (Genechem Co. Ltd., Shanghai, China). All the viral vectors contained GFP as a marker to track lentivirus-mediated target gene expression by fluorescence microscopy. Briefly, pGC-LV-GV287-GFP-CLDN7 or a control vector mixed with pHelper1.0 and pHelper2.0 were cotransfected into HEK-293 T cells with PEI (Sigma-Aldrich). Caki-1 and A498 cells were infected via lentiviruses according to the MOI value (the number of lentiviruses per number of cells) recommended by the manufacturer. Caki-1 or A498 CLDN7 and Caki-1 or A498 CTRL cells were sorted using a flow cytometer sorter (BD FACS AriaTM SORP, New Jersey, USA). The Luciferase-pcDNA3 was a gift from William Kaelin (Addgene plasmid # 18964) [13]. Lentiviruses were produced using a three-vector system: Luciferase-pcDNA3: viral packaging (psPAX2): viral envelope (pMD2G) at a 4:3:1 ratio in HEK-293 T cells. Caki-1 CLDN7-Luc and Caki-1 CTRL-Luc cells were selected with neomycin (200 μg/mL). The transfection efficiency was validated (Additional file 2: Figure S1).

### Quantitative real-time PCR (qRT-PCR) and reverse transcription PCR (RT-PCR)

Total RNA was extracted from the tissue samples or the transfected cells using the TRIzol reagent (Invitrogen; Thermo Fisher Scientific Inc.), according to the manufacturer’s instructions. cDNA was generated using reverse transcription (TansGEN, Beijing, China). qRT-PCR was performed using the ABI PRISM 7000 Fluorescent Quantitative PCR System (Applied Biosystems, Foster City, CA, USA), according to the manufacturer’s instructions, and normalized to GAPDH*.* RT-PCR was performed by electrophoresis on a 1.5% agarose gel. All experiments were repeated at least three times. The detailed primer sequences included in this study are shown in Additional file 3: Table S2.

### Immunohistochemistry (IHC) and Western blot analysis

The immunohistochemistry (IHC) and IHC scoring were carried out according to protocols that have been described previously [14]. Protein lysates were prepared by homogenization in 1% NP40 containing 1 mM PMSF and 20 μg protein was separated by SDS-PAGE. The immunoreactive bands were visualized using an Immobilon™ Western Kit (Millipore, Billerica, MA) using the SYNGENE G: BOX imaging system (Frederick, USA). Antibodies specific to CLDN7 (ab27487), BCL-2 (ab32124), PARP1 (ab32064), Caspase-3 (ab13847), E-cadherin (CDH1, ab76055), N-cadherin (CDH2, ab18203), Vimentin (ab92547) and TGFB1 (ab25121) were purchased from Abcam (Hong Kong, China). GAPDH (TA309157) and Ki-67 (TA500265) were purchased from ZSGB-BIO, Beijing, China. Cleaved-Caspase3 (Asp175) (9661S) was purchased from Cell Signaling Technology, MA, USA. The antibodies for the IHC and Western blot assays were diluted with phosphate buffered solution (PBS) or PBS plus Tween-20 (PBST), according to the manufacturer’s instructions.

### Methylation-specific PCR (MSP) and bisulfite genomic sequencing PCR (BGS)

Genomic DNA (1 μg) was denatured using 0.3 M NaOH for 10 min at 37 °C. The samples were then incubated at 50 °C for 16 h after adding hydroquinone (Sigma-Aldrich, St. Louis, Missouri, USA) and sodium bisulfate (Sigma-Aldrich). Genomic DNA was analyzed via MSP using primer sets located within a CpG-rich area in the CLDN7 promoter. PCR samples were then resolved by electrophoresis on a 1.5% agarose gel. For the BGS assay, DNA was purified, and a CpG-rich promoter region was amplified by PCR. The PCR products were purified and cloned into a PCR 2.1-TA cloning vector (Invitrogen). A minimum of six positive clones from each product were selected for sequencing. The detailed primer sequences are shown in Additional file 3: Table S2.

### Demethylation analysis

Caki-1 and A498 cells were seeded in six-well plates at a concentration of 1 × 10^5^ cells per well, grown for 24 h, and then treated with 5 μM 5-Aza-2′-deoxycytidine (5-Aza-dC, A, Sigma-Aldrich) for 4 days. Cells were cultured with or without 100 nM Trichostatin A (TSA, T, Sigma-Aldrich) for the final 24 h. RNA was isolated for RT-PCR analysis and DNA was extracted for CLDN7 MSP.

### MTS assay

The metabolic activity of Caki-1 cells was assessed using CellTiter 96™ AQueous Nonradioactive Cell Proliferation Assay, according to the manufacturer’s instructions (Promega, Madison, WI, USA). The optical density of the wells was measured at 450 nm using a Multiscan microplate spectrophotometer (Thermo LabSystems, Milford, MA, USA).

### Ethynyl-2-deoxyuridine (EdU) incorporation assay

Cell proliferation was also determined by Ethynyl-2-deoxyuridine incorporation assay using an EdU Apollo DNA in vitro kit (RIBOBIO, Guangzhou, China), according to the manufacturer’s instructions. Experiments were repeated at least three times.

### Flow cytometry analysis assay

Cell apoptosis was assayed by staining with Annexin V-APC and PI (KeyGEN BioTECH) following manufacturer’s instructions and detected by a flow cytometer (FACSCalibur, Becton Dickinson, New Jersey, USA).

### Wound healing assays

Cell migration was determined via a wound-healing assay. Briefly, approximately 3 × 10^5^ cells were seeded in 12-well plates at equal densities and grown to 80%~ 90% confluency. Artificial gaps were generated by a 200 μL sterile pipette tip after transfection. Wounded areas were marked and photographed with a microscope (Leica DM IL, Leica Microsystems, Germany) equipped with a digital camera (Leica DFC300FX).

### Transwell migratory and invasive assays

For the transwell migration assay, 1000 cells were plated into the upper chambers (24-well insert, pore size 8 μm, Corning) with 100 μL serum-free PRIM-1640. The lower chambers were filled with 500 μL PRIM-1640 containing 10% fetal bovine serum. Two days later, cells under the surface of the lower chamber were washed with PBS and stained with 0.5% crystal violet for 30 min.

For the invasion assay, 2000 cells were seeded on transwells (24-well insert, pore size 8 μm, Corning) coated with 60 μL Matrigel (1:3 dilution in PBS, Product #354234, Corning Inc., NY, USA). The culture conditions were the same as described for the transwell migration assay. After 72 h, adherent cells on the lower surface were stained with 0.5% crystal violet. The number of cells on the lower surface was photographed with a microscope (Leica DM IL, Leica Microsystems) equipped with a digital camera (Leica DFC300FX).

### Mouse model experiments

Animal experiments were conducted in accordance with the NIH Guidelines for the Care and Use of Laboratory Animals with the approval of the Review Board of Peking University First Hospital, Beijing. Mice were maintained under pathogen-free conditions with regulated temperature and humidity levels. Mice were randomly assigned to cages in groups of 5 and fed ad libitum under controlled light/dark cycles.

Twenty 5-week-old male BALB/c nude mice were purchased from Vitalriver, Beijing, China. Approximately 5 × 10^6^ Caki-1 CLDN7 or Caki-1 CTRL cells suspended in 100 μL Hank’s Balanced Salt Solution (Thermo Fisher Scientific Inc., USA) were mixed with Matrigel (1:1, Product #354234, Corning Inc., NY, USA). Then, 200 μL tumor cells were subcutaneously implanted on the right flank of 6-week BALB/c nude mice using a 28-gauge needle (Thermo Fisher Scientific Inc., USA). Tumor size was measured every fourth day and calculated using the formula: (length × width^2^)/2.

For the metastasis experiment, eight 5-week-old male B-NDG mice (NOD- *Prkdc*^*scid*^ *IL2rg*^*tm1*^/Bcgen) that lacked mature T cells, B cells, and natural killer (NK) cells, were purchased from BIOCYTOGEN, Beijing, China. Approximately 1 × 10^6^ Caki-1 CTRL-Luc or Caki-1 CLDN7-Luc cells were suspended in 200 μL PBS and injected into the lateral tail veins of each unanesthetized B-NDG mouse at six-weeks-old. Twenty days after injection, mice were anesthetized with isoflurane (YIPIN Pharmaceutical CO., LTD, Hebei, China). Ten minutes after D-Luciferin, sodium salt (150 mg/kg) was injected intraperitoneally, and cancer cells were detected with an in vivo imaging system, Xenogen IVIS (PerkinElmer, MA, USA). The total flux in photons per second were calculated and reported for each mouse’s lung and liver region using Living Image 4.3.1 (PerkinElmer/Caliper).

### High-throughput cDNA sequencing (RNA-Seq)

The RNA-Seq experiments were performed by Novogene (Beijing, China). The RNA-seq library was prepared for sequencing using standard Illumina protocols. Briefly, total RNAs from Caki-1 CTRL and CLDN7 cells were isolated using TRIzol reagent (Invitrogen) and treated with RNase-free DNase I (New England Biolabs, MA, USA), to remove any contaminating genomic DNA. RNA extraction was performed using Dynabeads oligo(dT) (Invitrogen Dynal). Double-stranded complementary DNAs were synthesized from 1 μg of total RNA using Superscript II reverse transcriptase (Invitrogen) and random hexamer primers. *Escherichia coli* RNase H (New England Biolabs) were added to remove RNA complementary to the cDNA. The cDNA was then fragmented by nebulization, and the standard Illumina protocol was followed thereafter to create the mRNA-seq library. The libraries were sequenced on an Illumina HiSeq 2000 platform. Sequencing reads were aligned to the human genome (hg19) using the TopHat program (v2.1.1) set to the default parameters. Total read counts for each protein-coding gene were extracted using HTSeq (version 0.6.0) and then loaded into R package DESeq2 to calculate the differentially expressed genes with a cut-off fold change of ≥1.5 and an FDR < 0.05. Gene expression was calculated using the RPKM (reads per kilobase transcriptome per million reads) method. Experiments were repeated three times.

### Statistical analyses

All data were expressed as means ± SD from at least three separate experiments. All data were analyzed using SPSS 20.0 statistical software (IBM. Chicago, IL, USA). The comparison of immunostaining intensities between the two groups was analyzed using a Mann-Whitney U test. The clinicopathological correlation with gene expression of the patients was analyzed using a chi-square test. Survival curves for patients were plotted using the Kaplan-Meier method, with log-rank tests for statistical significance. Uni- and multivariable Cox regression analyses were used to test the prognostic relevance between clinicopathological and immunohistochemical data. In case of multiple tests, a one-way ANOVA followed by Bonferroni-Holm procedure was applied. Other statistically significant differences were determined using Student’s T-test. Statistical significance was defined as a two-tailed *p* < 0.05. *p < 0.05, **p < 0.01.

## Results

### CLDN7 downregulation was associated with poor prognostic features

**Fig. 1:**
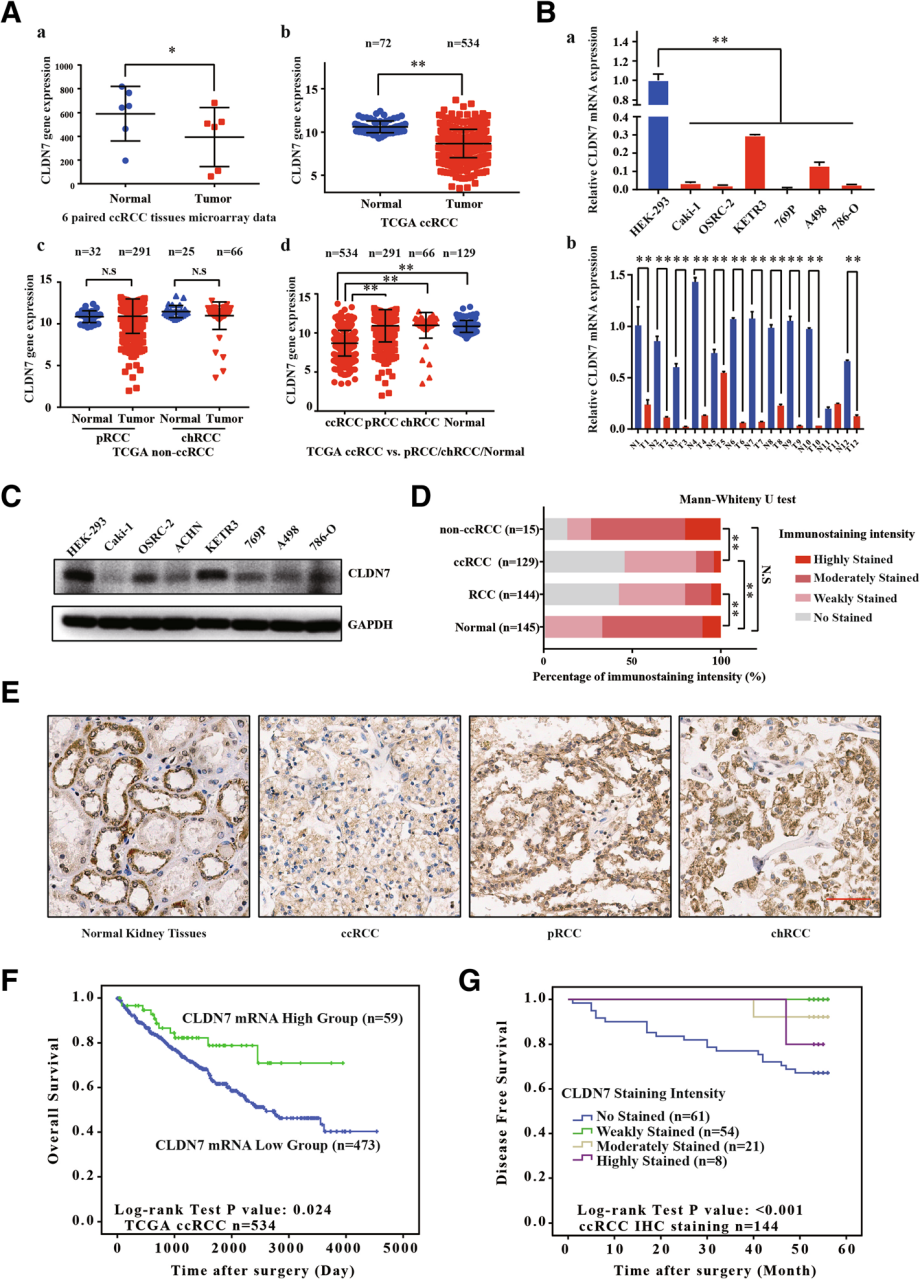
CLDN7 was downregulated in ccRCC patients and associated with poor prognosis. (**A**) *a*. Microarray chip assay showed CLDN7 expression in 6 ccRCC tissues and paired adjacent normal kidney tissues from Peking University First Hospital. *b*. Comparison of CLDN7 mRNA expression between normal kidney and ccRCC tissues. *c*. Comparison of CLDN7 mRNA expression between normal kidney and non-ccRCC tissues. *d*. Comparison of CLDN7 mRNA expression among normal kidney, ccRCC, chRCC and pRCC tissues. (**B**) *a*. Relative expression of CLDN7 mRNA in ccRCC cell lines compared with normal kidney cell HEK-293. *b*. Relative expression of CLDN7 mRNA in 12 paired ccRCC tissues and adjacent normal kidney tissues. (**C**) Western blot showed CLDN7 protein expression in RCC cell lines and HEK-293. (**D**) Immunohistochemistry staining analysis of CLDN7 protein expression in 144 RCC tissues and adjacent normal kidney tissues (tissue microarray, total 129 ccRCC, 15 non-ccRCC and 145 normal kidney tissues). Immunostaining intensity was graded as no stained, weakly stained, moderately stained and strongly stained. (**E**) The representative immunostaining of CLDN7 in normal kidney, ccRCC, pRCC and chRCC tissues (Scale bar, 100 μm). (**F**) Kaplan-Meier curves of overall survival time between low (*n* = 473) and high (*n* = 59) CLDN7 mRNA expression group in 534 ccRCC patients from TCGA. The cut-off value was the mean of CLDN7 mRNA expression in 129 normal kidney tissues. (**G**) Kaplan-Meier curves of disease free survival time in 4 respective CLDN7 protein expression group including No stained (*n* = 61), Weakly stained (*n* = 54), Moderately stained (*n* = 21) and Strongly stained (*n* = 8) in 144 RCC patients from our hospital. Results that were expressed as the mean ± SD from at least three independent experiments. N.S refers to not significant. * refers to *p* < 0.05. ** refers to *p* < 0.01

Our previous microarray chip assay of 6 ccRCC tissues and paired adjacent normal kidney tissues showed that CLDN7 was significantly downregulated in ccRCC tissue compared with normal kidney tissue (Fig. 1A a). To determine the expression of CLDN7, we analyzed the RNA-Seq data of RCC cases from TCGA. CLDN7 mRNA expression was decreased significantly in ccRCC tissues, but not in chRCC and pRCC tissue, compared to adjacent normal kidney tissues (Fig. 1A b, c, d). Next, we performed qRT-PCR in the RCC cell lines and 12 paired human ccRCC tissues. CLDN7 mRNA expression was significantly lower in the RCC cells and ccRCC tissues than in the human embryonic kidney cells (HEK-293) and adjacent normal kidney tissues (Fig. 1b). Western blots produced similar results, with CLDN7 protein being downregulated in the RCC cell lines (Fig. 1c). Immunostaining of the 144 RCC tissues and adjacent normal kidney tissues also showed that intensity of immunostaining was significantly weaker in the ccRCC tissues compared with their adjacent normal tissues (Fig. 1d). Moreover, CLDN7 staining intensity was lower in ccRCC tissue than in non-ccRCC tissue. The representative immunostaining of CLDN7 in normal kidney tissues, ccRCC, pRCC and chRCC tissues are shown in Fig. 1e.

To explore the correlation between CLDN7 expression and clinicopathological features, we divided the TCGA 534 ccRCC patients into different groups based on the mean value (10.625 FPKM) of CLDN7 mRNA expression of the adjacent normal kidney tissues. The results showed that CLDN7 mRNA level was strongly correlated with gender, pathologic M, pathologic T, pathologic stage and overall survival (Additional file 4: Table S3).

We further analyzed the CLDN7 immunostaining intensity with clinicopathological parameters in 129 ccRCC patients from Peking University First Hospital. The results showed that the CLDN7 protein level was strongly correlated with age, pathologic T, histologic grade and risk of recurrence, metastasis and death after surgery (Table 1). Collectively, CLDN7 was downregulated in ccRCC patients and was significantly associated with tumor progression features.

**Table 1:** Correlation between CLDN7 immunostaining intensity and clinicopathological features in 129 ccRCC patients from Peking University First Hospital (*, *p* < 0.05. **, *p* < 0.01)

Clinicopathological features		CLDN7 immunostaining intensity				*p* value
		0	1	2	3	
Gender	Female	38	39	8	2	0.318
	Male	21	13	5	3	
Age(median 55, year)	<=55	34	29	3	0	0.013*
	> 55	25	23	10	5	
Laterality	Left	24	27	4	4	0.176
	Right	35	25	9	1	
Pathologic T	T1	47	44	9	1	0.001**
	T2	2	5	1	0	
	T3	10	3	3	4	
Histologic grade	G1	10	6	1	0	0.044*
	G2	39	40	7	2	
	G3	7	5	5	3	
	G4	3	1	0	0	
DFS	Disease free	40	52	12	4	< 0.001**
	Recurrence, metastasis or death	19	0	1	1	
Creatinine	Normal	47	46	10	3	0.484
	Elevated	11	5	1	1	
Urea nitrogen	Normal	48	45	11	3	0.803
	Elevated	9	6	1	1	

*DFS* Disease Free Survival. Follow up (1–56 month). Recurrence 9, Metastasis 11 and Death 1

To explore the potential prognostic significance of CLDN7, we evaluated 534 ccRCC patients from TCGA using a Kaplan-Meier analysis with a log-rank test. As shown in Fig. 1f, the low CLDN7 mRNA expression group had a poorer prognosis than the higher CLDN7 group. Moreover, in 144 RCC patients from Peking University First Hospital, immunostaining results confirmed that ccRCC patients with low CLDN7 protein expression levels experienced recurrent or metastatic tumors earlier after surgery, compared to those with higher expression levels (Fig. 1g). Univariate and multivariate Cox regression analyses indicated that higher ages, pathologic T stage and elevated creatinine levels after surgery were unfavorable prognostic factors in ccRCC patients, except for high protein expression of CLDN7, which was a favorable prognostic factor (HR = 0.227, 95% CI = 0.093–0.551, *p* = 0.001) (Table 2). Therefore, decreased CLDN7 expression indicates poor prognosis in ccRCC patients.

**Table 2:** Uni- and multi-variate Cox regression of CLDN7 protein expression for disease free survival (DFS) in 129 ccRCC from Peking University First Hospital (*, *p* < 0.05. **, *p* < 0.01)

Factors	Univariate analysis		Multivariate analysis	
	HR (95% CI)	*p*	HR (95% CI)	*p*
CLDN7 Staining Intensity	0.254(0.101–0.639)	0.004**	0.227(0.093–0.551)	0.001**
3 vs. 2 vs. 1 vs. 0				
Gender	0.631(0.231–0.722)	0.368		
Female vs. Male				
Age (year)	1.754(0.727–4.233)	0.211	3.600(1.311–9.887)	0.013*
> 55 vs. <=55				
Laterality	0.906(0.385–2.133)	0.821		
Right vs. Left				
Pathologic T	2.175(1.386–3.413)	0.001**	2.419(1.551–3.773)	< 0.001**
T3 vs. T2 vs. T1				
Histologic grade	2.938(1.644–5.250)	< 0.001**		
G3 vs. G2 vs. G1				
Creatinine	0.254(0.101–0.639)	0.004**	5.809(2.184–15.454)	< 0.001**
Elevated vs. Normal				
Urea nitrogen	4.187(1.733–10.113)	0.001**		
Elevated vs. Normal				

### CLDN7 downregulation by promotor methylation in ccRCC was associated with adverse pathologic results and poor prognosis

**Fig. 2:**
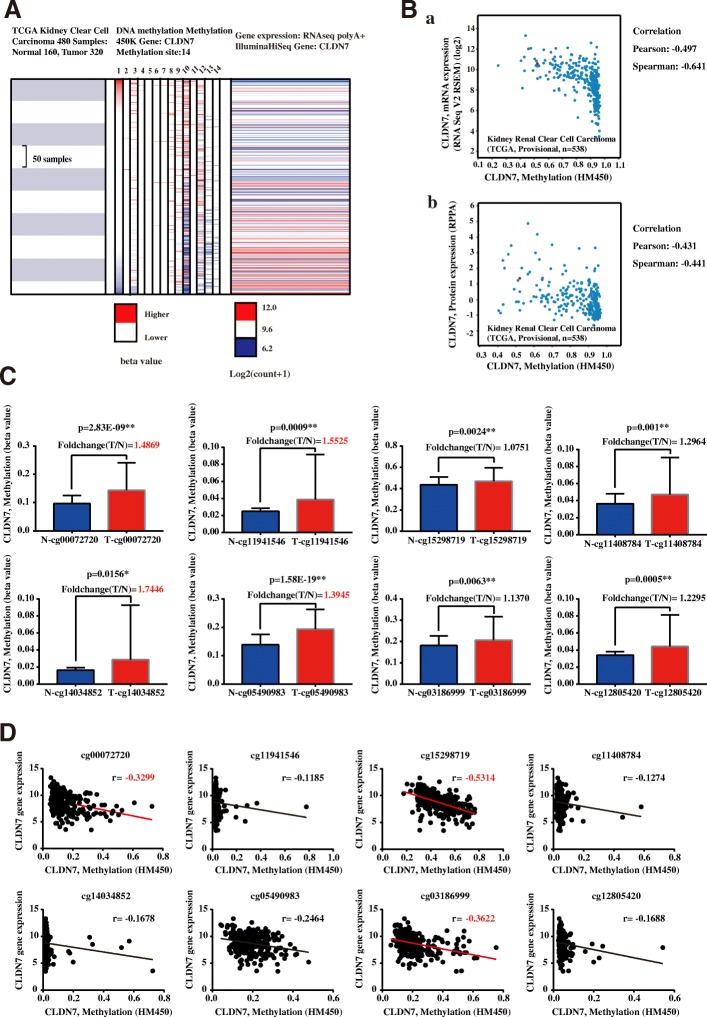
CLDN7 promoter DNA methylation is associated with low CLDN7 expression in ccRCC patients. (**A**) The heatmap between CLDN7 14 methylation region and CLDN7 mRNA expression in TCGA ccRCC dataset (*n* = 480). The analysis was performed using UCSC Xena. (**B**) The negative correlation between CLDN7 methylation status and CLDN7 mRNA (*a*) and protein expression (*b*) in Kidney Renal Clear Cell Carcinoma (TCGA, Provisional, *n* = 538) dataset, using cBioPortal. (**C**) Eight significant DNA hyper-methylated promoter regions (cg00072720, cg11941546, cg14034852, cg05490983, cg15298719, cg03186999, cg11408784 and cg12805420) of CLDN7 in ccRCC tissues compared with normal kidney tissues. The fold change (T/N) more than 1.30 were marked red. (**D**) Eight DNA hyper-methylated promoter regions (cg00072720, cg11941546, cg14034852, cg05490983, cg15298719, cg03186999, cg11408784 and cg12805420) of CLDN7, were negatively correlated with CLDN7 mRNA expression. The correlation coefficients lower than − 0.3 were marked red. T refers to Tumor tissue of ccRCC, N refers to Normal kidney tissue. *p < 0.05, **p < 0.01

Epigenetic alterations, such as promoter CpG methylation, could mediate the activation of oncogene and inactivate tumor suppressor genes, thus contributing to tumorigenesis [15]. CLDN7 promoter hypermethylation has been reported in several cancers [16–19]. In the TCGA ccRCC dataset, we generated a comparable heatmap in which we found that when some regions of the CLDN7 promoter were hypermethylated, CLDN7 expression was lower (Fig. 2a). Moreover, we observed that CLDN7 methylation status was negatively related to its mRNA and protein expression in the TCGA ccRCC dataset (Fig. 2b). Collectively, CLDN7 downregulation in ccRCC was significantly associated with promoter hypermethylation.

Furthermore, we analyzed the 14 methylation regions of CLDN7 in the TCGA datasets (UCSC Xena). The results suggested that most of the methylation regions of CLDN7 (8/14) were significantly hypermethylated in ccRCC tissues compared with adjacent normal tissues (Fig. 2c and Additional file 5: Figure S2). Among the four most hypermethylated CLDN7 promoter regions (cg00072720, cg11941546, cg14034852 and cg05490983), we found that the methylation status of cg00072720 was negatively correlated with CLDN7 mRNA expression and had the largest negative correlation coefficient (Fig. 2d and Additional file 6: Figure S3). Taken together, we chose to use cg00072720 in the following experiments.

To explore the clinical application of CLDN7 methylation status, we performed methylation-specific PCR analyses in 108 ccRCC tissues from Peking University First Hospital. The relationship between CLDN7 methylation status and clinicopathologic features are summarized in Table 3. Hypermethylated CLDN7 was significantly correlated with advanced age, pathologic T and histologic G, which agrees with our bioinformatic analysis of the TCGA ccRCC Methylation 450 K dataset (*n* = 319, Additional file 7: Table S4). Interestingly, the CLDN7 promoter hypermethylated group had a poor prognosis (Additional file 8: Figure S4). Therefore, hypermethylation of CLDN7 promoter was associated with downregulation of CLDN7 and a poor prognosis in ccRCC patients.

**Table 3:** The association between CLDN7 methylation status and clinicopathologic features in 108 ccRCC patients from Peking University First Hospital (*, *p* < 0.05. **, *p* < 0.01)

Clinicopathological Features	Number	CLDN7 Methylation Status		*p* Value
		Methylated	Unmethylated	
Overall	108	34 (31.5%)	74 (68.5%)	
Gender				
Male	73	26 (35.6%)	47 (64.4%)	0.181
Female	35	8 (22.9%)	27 (77.1%)	
Age (years)				
< 56 (Median)	59	12 (20.3%)	47 (79.7%)	0.006**
≥ 56	49	22 (44.9%)	27 (55.1%)	
Pathologic T				
T1a	51	13 (25.5%)	38 (74.5%)	0.038*
T1b	29	10 (34.5%)	19 (65.5%)	
T2	7	0	7 (100%)	
T3	21	11 (52.4%)	10 (47.6%)	
Hisologic grade				
G1	38	6 (15.8%)	32 (84.2%)	0.032*
G2	59	23 (39.0%)	36 (61.0%)	
G3	11	5 (45.5%)	6 (54.5%)	

### Promoter hypermethylation contributed to downregulation of CLDN7 in ccRCC

**Fig. 3:**
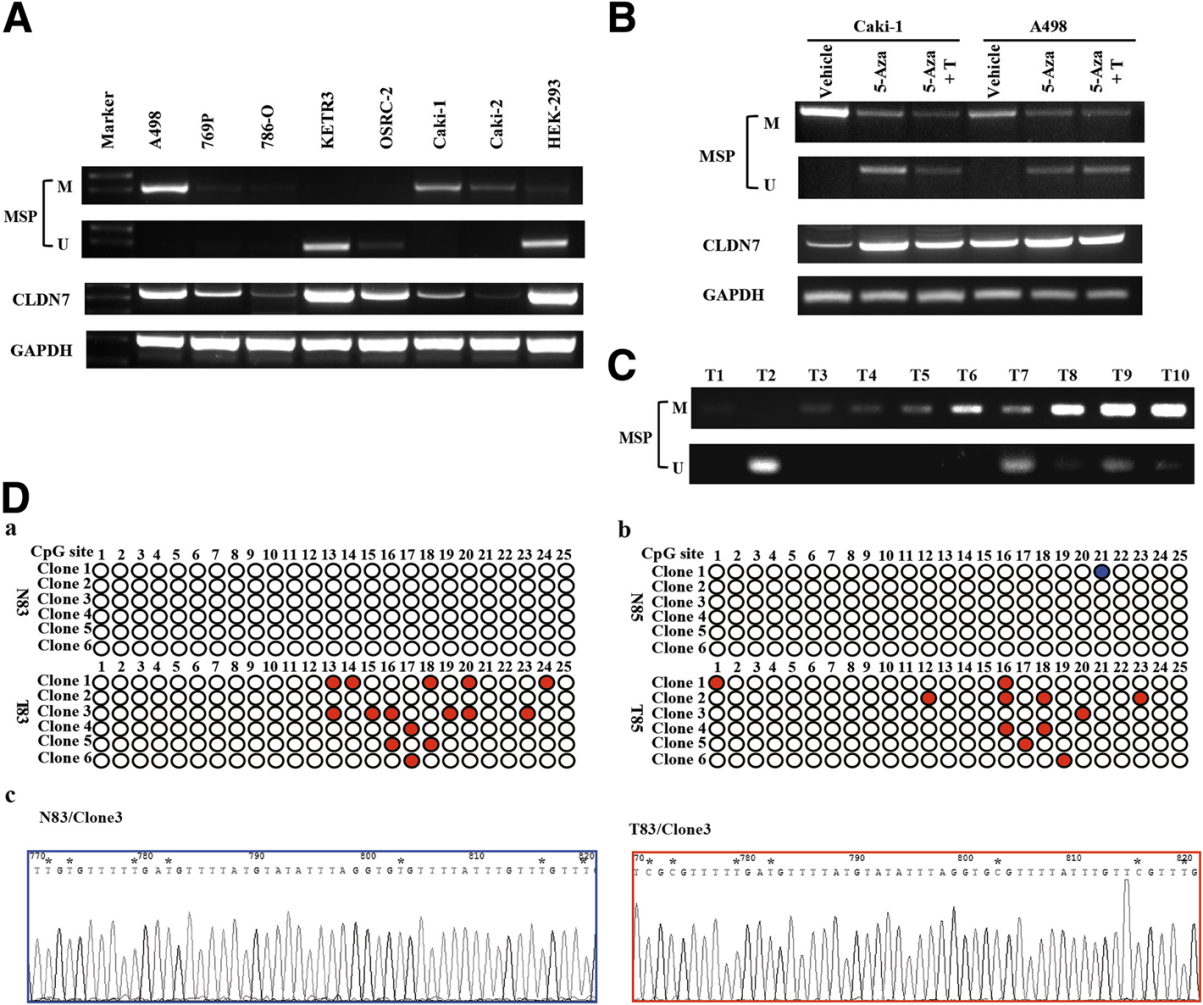
Promoter hyper-methylation contributes to downregulation of CLDN7. (**A**) MSP analysis of CLDN7 promoter DNA methylation status and RT-PCR analysis of CLDN7 mRNA expression in RCC cell lines. (**B**) Treatment with 5-aza-dC and TSA demethylated CLDN7 promoter and increased CLDN7 mRNA expression in Caki-1 and A498 cells. (**C**) The representative MSP analysis of CLDN7 promoter DNA methylation status in ccRCC tissues. (**D**) Representative results of bisulfite genomic sequencing analysis of DNA methylation status of CLDN7 promoter in two ccRCC patients (*a*. No.83 and *b*. No.85). DNA methylation status of the CLDN7 promoter in blue (normal) or red (tumor) filled circles indicate DNA methylation, open circles indicate absence of DNA methylation. Representative bisulfite genomic sequencing results of normal kidney tissues (*c*) and ccRCC tissues (*d*) in No.83 ccRCC patient. * indicate the CpG islands. T refers to Tumor tissue of ccRCC, N refers to Normal kidney tissue. U = Unmethylated, M = methylated. 5-Aza refers to 5-Aza-2′-deoxycytidine. T refes to Trichostatin A

As shown in Fig. 2, promoter hypermethylation was associated with low CLDN7 expression in ccRCC patients. We designed primers for the cg00072720 region that was mapped to the CLDN7 promoter for experimental validation. The results suggest that RCC cells had low mRNA levels of CLDN7, which is in accordance with their high hypermethylation (Figs. 1b and 3a). The CLDN7 promoter was hypermethylated in both Caki-1 and A498 cells. Treatment with 5-Aza-2′-deoxycytidine (5-aza-dC, 5-Aza, A) and Trichostatin A (TSA, T) induced demethylation of the CLDN7 promoter and increased CLDN7 mRNA expression (Fig. 3b). CLDN7 hypermethylation was also found in 34/108 (31.5%) ccRCC tissues (Fig. 3c and Table 3). Since MSP analyzed only a few CpG sites in the promoter, we sequenced the CLDN7 promoter region described above. Sequencing of sodium-bisulfite-treated DNA from two ccRCC patients (No. 83 and 85) showed hypermethylation of CpG sites in ccRCC tumor tissues (T) compared with adjacent normal kidney tissues (N) (Fig. 3d).

### CLDN7 overexpression attenuated tumor proliferation and induced tumor apoptosis in metastatic ccRCC cells

**Fig. 4:**
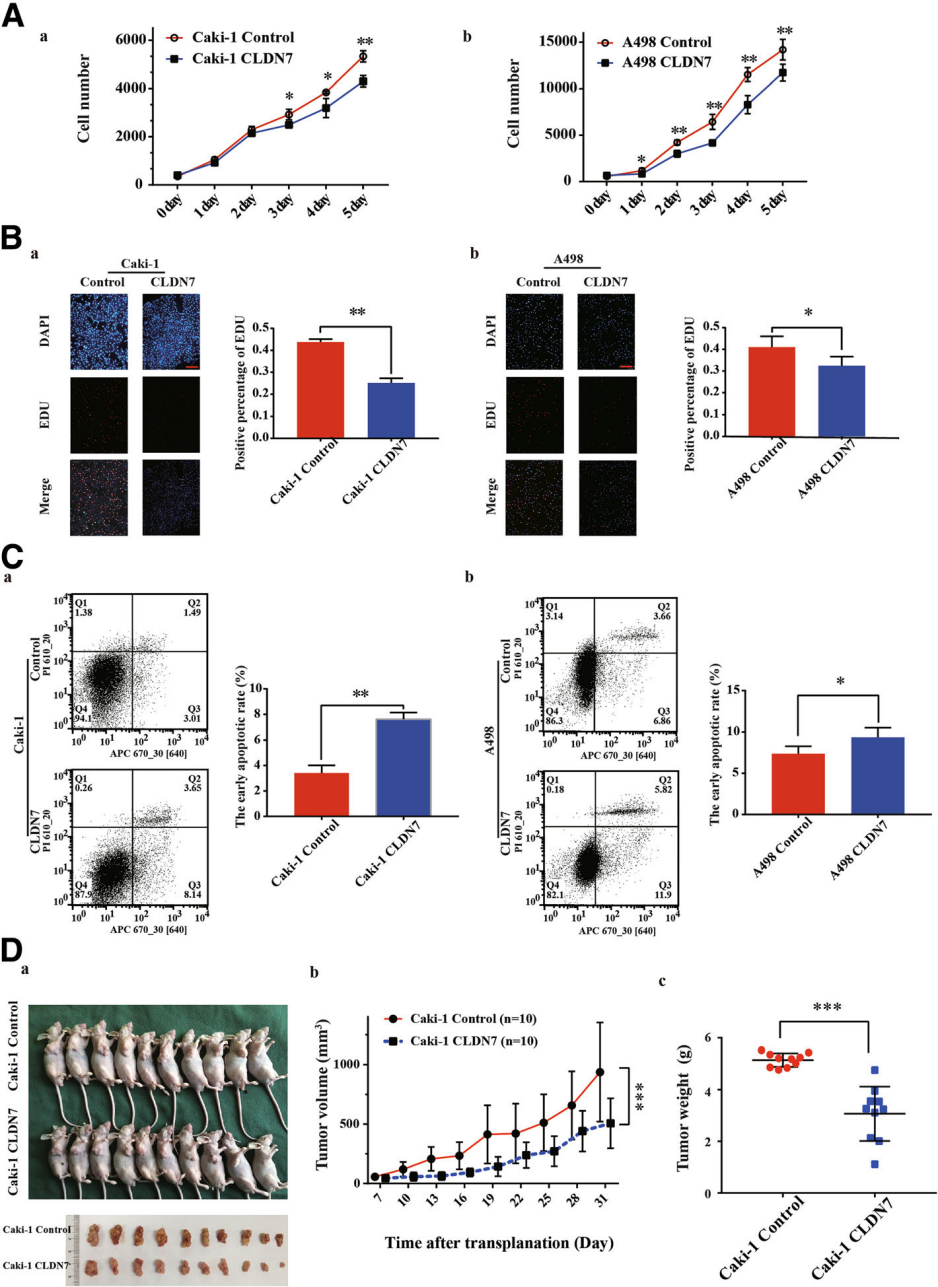
CLDN7 overexpression attenuated tumor proliferation and induced tumor apoptosis in vitro and in vivo. (**A**) MTS assay determined cell proliferation of Caki-1 CLDN7 (*a*) and of A498 CLDN7 (*b*) comparing with control cells at indicated time. (**B**) *a.* The representative images of Caki-1 CLDN7 and Caki-1 Control cells by EDU incorporation assay. Scale bar, 100 μm (left panel). Statistical analysis of cell proliferation ability of Caki-1 CLDN7 and Caki-1 Control cells by positive percentage of EDU (right panel). *b.* Representative images of EDU incorporation assay in A498 cells. Scale bar, 100 μm. Statistical analysis of cell proliferation ability of A498 CLDN7 and A498 Control cells by positive percentage of EDU. (**C**) *a.* Representative images of Caki-1 CLDN7 and Caki-1 Control cells stained with APC and PI measured by flow cytometer (left panel). Statistical analysis of early cell apoptosis of Caki-1 CLDN7 and Caki-1 Control cells (right panel). *b.* Representative images of A498 CLDN7 and A498 Control cells stained with APC and PI measured by flow cytometer (left panel). Statistical analysis of early cell apoptosis of Caki-1 CLDN7 and Caki-1 Control cells (right panel). (**D**) Mice experiment. *a.*. Images of 20 mice and xenografts formed by Caki-1 Control and Caki-1 CLDN7 cells one month after planation. *b.* and *c.* Statistical analysis of growth curves and tumor weights of xenografts derived from Caki-1 Control and Caki-1 CLDN7 cells. Data were presented as mean ± SD from at least three independent experiments. *p < 0.05, **p < 0.01

Lower expression and higher methylation of CLDN7 were observed in Caki-1 and A498 cells. Therefore, we generated a GFP labeled, lentiviral vector-expressing control and CLDN7-expressing Caki-1 and A498 cells for a functional study. The effect of CLDN7 on the growth of Caki-1 and A498 cells was determined using MTS assays and an EdU incorporation assay. The results suggested that overexpression of CLDN7 inhibited cell proliferation in Caki-1 and A498 cells (Fig. 4a, b).

Cell proliferative alteration is usually caused by changes in the cell cycle or apoptosis. Flow cytometry was performed to evaluate the early apoptosis in the cells. Overexpression of CLDN7 induced significant apoptosis in Caki-1 and A498 cells (Fig. 4c). Consistent with the weak proliferative ability and high apoptotic rate observed in the CLDN7 overexpressed Caki-1 and A498 cells, a xenograft experiment in mice found that CLDN7 overexpressed tumors grew slower than those in a control group (Fig. 4d).

### CLDN7 overexpression significantly decreased cell migratory and invasive ability

**Fig. 5:**
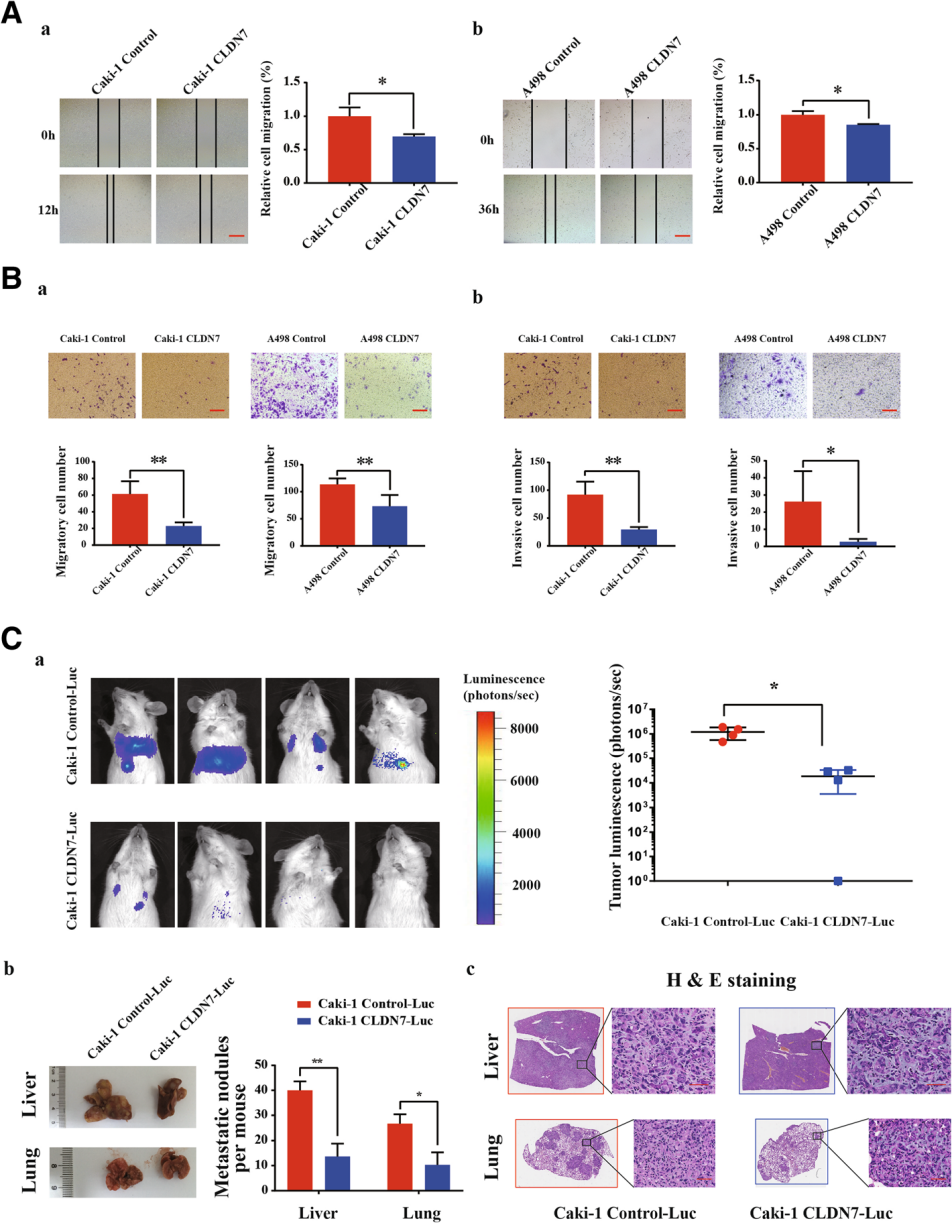
CLDN7 overexpression significantly decreased cell migratory and invasive ability in vitro and in vivo. (**A**) Wound healing assay. *a.* Left panel showed the representative images of wound of Caki-1 CLDN7 and Control cells at 0 h and 12 h. Scale bar, 100 μm. Relative cell migratory distance of Caki-1 CLDN7 and Control were statistically analyzed (right panel). *b*. Representative images of wound of A498 CLDN7 and Control cells at 0 h and 36 h. Scale bar, 100 μm (Left panel). Relative cell migratory distance of A498 CLDN7 and Control were statistically analyzed (right panel). (**B**) Transwell migrantion and invasion assay. *a*. Representative images of transwell migration assay of Caki-1 and A498 CLDN7 and Control cells for 48 h. Scale bar, 100 μm (Top). Statistically analysis of migratory cells from Caki-1 and A498 CLDN7 and Control cells (bottom). *b.* Representative images of transwell invasion assay of Caki-1 and A498 CLDN7 and Control cells for 72 h. Scale bar, 100 μm (Top). Statistically analysis of invasive cells from Caki-1 and A498 CLDN7 and Control cells (bottom). (**C**) Mouse metastasis model. *a.* Representative images of metastatic lesions derived from Caki-1 Control and CLDN7 cells 20 days after transplanation. It is detected by in vivo imaging system-Xenogen IVIS. Statistical analysis of tumor luminescence (photons/ sec) between Caki-1 Control-Luc and Caki-1 CLDN7-Luc group (bottom panel). *b*. Representative images of liver (top) and lung (bottom) with metastatic lesions derived from Caki-1 Control-Luc and CLDN7-Luc cells (left panel). Right panel showed the statistical analysis of metastatic nodules in liver and lung per mouse. *c*. Representative HE staining of liver (top) and lung (bottom) with metastatic lesions derived from Caki-1 Control-Luc and CLDN7-Luc cells. Scale bar, 100 μm. *p < 0.05, **p < 0.01, Student’s Test

The migratory and invasive abilities of CLDN7 overexpressed Caki-1 and A498 cells were significantly decreased, compared with control cells. The results showed that migratory distances of the CLDN7 overexpressed group were significantly inhibited (Fig. 5a). A transwell migratory and invasive assay also found that migratory and invasive cells were clearly attenuated in Caki-1 and A498 cells that overexpressed CLDN7, compared with control cells (Fig. 5b). To confirm the inhibitory effect of CLDN7 on cell migration and invasion, an in vivo model of metastasis was established. Metastasis experiments indicated that overexpression of CLDN7 inhibited metastasis to liver and lung (Fig. 5c). The number and size of metastatic lesions formed by Caki-1 CLDN7-Luc cells were significantly fewer and smaller than the metastatic lesions formed by Caki-1 Control-Luc cells (Fig. 5c).

### Mechanisms of tumor suppression by CLDN7 in ccRCC

To understand the tumor suppressive role of CLDN7 in ccRCC, gene-set enrichment analysis on the data from the database of TCGA was used to compare the differences in cell processes between the low- and high- CLDN7 groups. Interestingly, the results suggest that several pathways relating to cancer and TGF-β, WNT, Focal adhesion and NOTCH pathways relating to epithelial-mesenchymal transition (EMT) and tumor progression were all decreased in ccRCC with high CLDN7 mRNA expression (Additional file 9: Figure S5 and Additional file 10: Table S5).

**Fig. 6:**
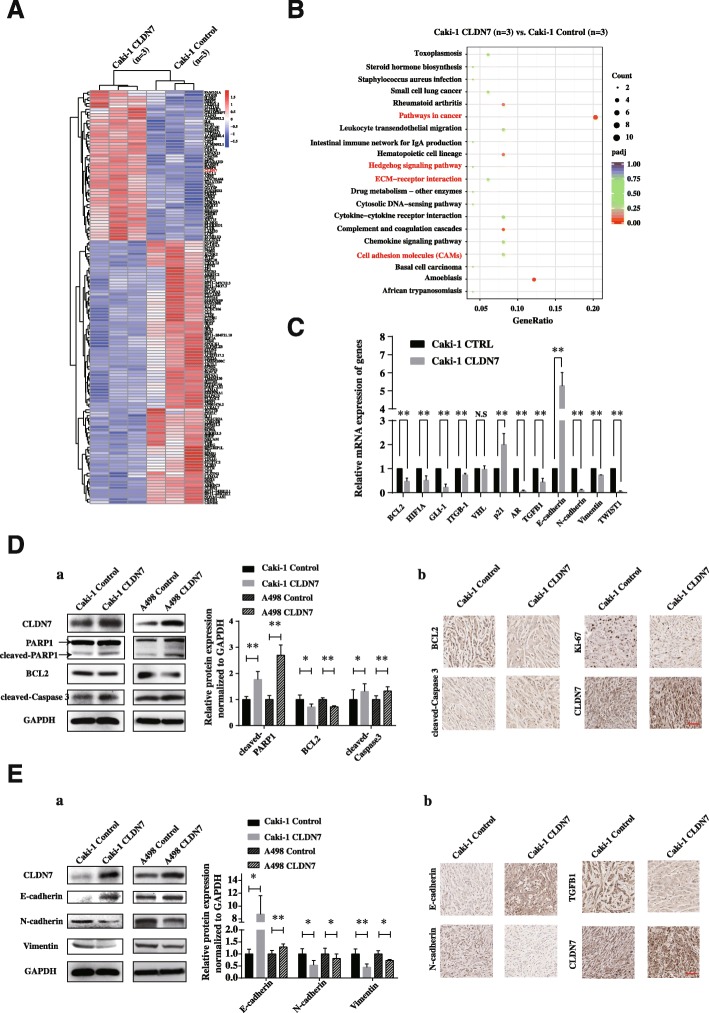
Mechanism of tumor suppressive function of CLDN7 in ccRCC. (**A**) Heatmap representation of differentially expressed genes identified by RNA-Seq between Caki-1 CLDN7 cells (*n* = 3) and Caki-1 Control cells (n = 3). (**B**) Statistics of KEGG pathway enrichment. The y-axis corresponds to KEGG Pathway, and the x-axis shows the GeneRatio. The color of the dot represent adjusted *p* value (padj), and the size of the dot represents the number of differentially expressed genes mapped to the reference pathways. (**C**) Validation of differentially expressed genes by qRT-PCR. Comparison of mRNA expression of genes in pathways of cancer (*BCL2*, *HIF1A*, *GLI-1*, *ITGB-1*, *p21* and *AR*) and genes in EMT-related pathway (*TGFB1*, *E-cadherin*, *N-cadherin*, *Vimentin* and *TWIST1*) between Caki-1 CLDN7 cells and Caki-1 Control cells. All data are shown as means ± SD. (**D**) *a*. Western blot assay (left) and statistical analysis (right) of CLDN7, BCL2, cleaved-PARP1 and cleaved-Caspase 3 expression in Caki-1 and A498 cells, while overexpression of CLDN7 compared with control group. *b*. IHC assay of BCL2, cleaved-Caspase 3, Ki-67 and CLDN7 expression in xenografts formed by Caki-1 CLDN7 and Control cells. Scale bar, 100 μm. (**E**) *a*. Western blot assay and statistical analysis of E-cadherin, N-cadherin and Vimentin expression in CLDN7 overexpressed Caki-1 and A498 cells, comparing with control cells. *b*. IHC assay of E-cadherin, N-cadherin, TGFB1 and CLDN7 in xenografts formed by Caki-1 CLDN7 and Control cells. Scale bar, 100 μm. N.S, not significant. *p < 0.05, **p < 0.01, Student’s Test

To confirm these results, we performed mRNA sequencing in Caki-1 CLDN7 and control cells, which suggested that many genes are differently expressed when CLDN7 is overexpressed. These differently expressed genes could separate CLDN7 overexpressed Caki-1 cells from control cells (Fig. 6a). Furthermore, a Kyoto Encyclopedia of Genes and Genomes (KEGG) pathway analysis found that genes influenced by CLDN7 overexpression were mostly enriched in pathways involved with cancer and cell adhesion molecules (CAMs), extracellular matrix (ECM)-receptor and Hedgehog signaling pathways that relate to EMT and tumor progression (Fig. 6b). These genes screened by RNA-Seq were validated by qRT-PCR. The results showed that oncogenes (*BCL2*, *HIF1A*, *GLI-1*, *ITGB-1* and *AR*) were significantly downregulated, while tumor suppressor gene *p21* was significantly upregulated in CLDN7 overexpressed Caki-1 cells. Additionally, the epithelial marker, *E-cadherin*, was increased while mesenchymal markers, *N-cadherin* and *Vimentin* were decreased. This finding suggests that there is a mesenchymal-epithelial transition (MET) in CLDN7 overexpressed cells. Interestingly, the EMT inducers, *TGFB1* and *TWIST1* were both downregulated by CLDN7 (Fig. 6c). Cell pro-apoptotic markers, such as cleaved-PARP1 and cleaved-Caspase3, were upregulated by CLDN7 in Caki-1 and A498 cells, and the anti-apoptotic marker BCL2 was downregulated (Fig. 6d). Moreover, BCL2 and the cell proliferation marker Ki-67 were suppressed by CLDN7 while cleaved-Caspase3 was upregulated in xenograft tissues (Fig. 6d). To validate the mechanism of CLDN7 in cell adhesion and metastasis, EMT markers were evaluated in cultured cells and cell derived xenografts. It was found that CLDN7 overexpression promoted the expression of E-cadherin in vitro and in vivo. In contrast, expression of TGFB1, N-cadherin and Vimentin were all downregulated by CLDN7 (Fig. 6e). Taken together, CLDN7 suppressed cell growth and metastasis by inducing cell apoptosis and inhibiting EMT.

## Discussion

Previous studies have reported that CLDN7 is dysregulated in various cancers [10–12, 18–23]. During our study, we confirmed that there is a downregulation of CLDN7 in ccRCC. Bioinformatic Data Mining found that DNA hypermethylation in the promoter of CLDN7, and MSP and BGS results confirmed the CLDN7 promoter hypermethylation in ccRCC tissues. Analysis of follow up data, clinical features and CLDN7 expression and methylation data, demonstrated that the lower expression and higher methylation status of CLDN7 were significantly associated with tumor progression and poor prognosis. Interestingly, in vitro and in vivo assays both found that CLDN7 overexpression significantly inhibited cell proliferation and induced apoptosis. Cell migratory and invasive abilities were also suppressed by CLDN7. To explore the molecular mechanism of the tumor suppressive function of CLDN7 in ccRCC, GSEA was performed to evaluate the different gene expression profiles between low- and high-CLDN7 expression groups of ccRCC patients. We found that cancer pathways and EMT-related pathways both decreased in ccRCC patients with high CLDN7 expression [23]. Furthermore, RNA-Seq found that different expressed genes influenced by CLDN7 overexpression were enriched in pathways related to cancer and EMT, which was confirmed by qRT-PCR, Western blot and IHC staining in vitro and in vivo. Taken together, it is suggested that CLDN7 may have a fundamental role in tumor progression in ccRCC, by downregulating genes in pathways relating to cancer and EMT at the transcriptional level.

Previous study has shown that TGFB1 exposure decreased expression of CLDN7 and diminished epithelial barrier function, however, CLDN7 overexpression resulted in protection from TGFB1-mediated barrier dysfunction [24]. Many transcription repressors, including TWIST1, ZEB-1, Musashi-2, and Snail, promote EMT and can bind to E-box motifs, thus suppressing CLDN7 expression [25–30]. CLDN7, as a member of tight junctions, is often considered to be downstream of EMT. Interestingly, a recent study reported that overexpression of CLDN7 in colon cancer induced epithelial features and suppressed EMT through upregulation of Rab25 then decreased expression of p-Src and mitogen-activated protein kinase–extracellular signal–regulated kinase 1/2 [19]. However, the present study found that in CLDN7 overexpressed ccRCC cells, Rab25 did not increased significantly. Notably, we discovered that CLDN7 downregulated TGF-beta signaling pathway significantly. Additionally, we found that the EMT inducer, TGFB1, was decreased by CLDN7. This result was validated in vitro and in vivo. Therefore, we propose a possible vicious circle between the loss of CLDN7 and upregulation of TGFB1 in ccRCC carcinogenesis and development. Further investigation of this cycle is part of the ongoing studies in our laboratory.

Finally, given the suppressive role of CLDN7 during the process of EMT induced by TGFB1 and Musashi-2 [24, 27], it is a novel strategy to inhibit tumor progression by increasing CLDN7 expression. Studies have demonstrated that CLDN7 overexpression inhibited human colon and lung cancer invasion though EMT and MAPK pathways [19, 31]. In addition, the EMT process itself has been shown to influence cellular resistance to a number of drugs [32]. And CLDN7 was revealed to increase chemosensitivity through the activation of caspase pathway in lung cancer [33]. In this study, we found that CLDN7 downregulation was associated with poorer prognosis and CLDN7 overexpression inhibited EMT-related pathways in ccRCC. Therefore, we propose that CLDN7 may serve as a biomarker or even a therapeutic target for ccRCC.

## Conclusions

In conclusion, this is the first report to show that downregulation of CLDN7 is closely associated with metastatic features and poor prognosis in ccRCC patients. We also proposed the mechanism that promoter hypermethylation contributes to downregulation of CLDN7 expression. Moreover, CLDN7 promoter hypermethylation is also related to adverse pathologic results and poor prognosis. CLDN7 overexpression could inhibit cell growth and metastasis of ccRCC cells in vitro and in vivo. Notably, we have demonstrated a previously undescribed and important role of CLDN7 in cancer-related and EMT-related pathways in ccRCC. Further investigations are required to confirm the potential clinical application of CLDN7 and elucidate the detailed molecular mechanisms between CLDN7 and TGFB1 in ccRCC.

## Additional files

Additional file 1: **Table S1.** The clinicopathological features of 120 ccRCC patients from Peking University First Hospital. (DOCX 14 kb)

Additional file 2: **Figure S1.** Transfection validation. (JPG 2066 kb)

Additional file 3: **Table S2.** The primers designed for this study. (DOCX 14 kb)

Additional file 4: **Table S3.** Correlation between CLDN7 expression and 653 clinicopathological features in 534 ccRCC patients from TCGA. (DOCX 15 kb)

Additional file 5: **Figure S2.** Six DNA methylation sites of CLDN7 promoter that not significantly hypermethylated in TCGA ccRCC dataset. (JPG 1171 kb)

Additional file 6: **Figure S3.** The six DNA methylation regions of CLDN7 promoter that are negatively correlated with CLDN7 mRNA expression in TCGA ccRCC dataset. (JPG 1164 kb)

Additional file 7: **Table S4.** Correlation between CLDN7 promoter DNA methylation site (cg00072720) and clinicopathological features in 319 ccRCC patients from TCGA. (DOCX 15 kb)

Additional file 8: **Figure S4.** The CLDN7 promoter DNA methylation site, cg00072720, was associated with poor overall survival time while in hypermethylated status. (JPG 470 kb)

Additional file 9: **Figure S5.** Gene-set enrichment analysis is used to identify the pathways in two different CLDN7 mRNA level groups. (JPG 2095 kb)

Additional file 10: **Table S5.** Gene-set enrichment analysis between high- and low- CLDN7 group in Kidney clear cell carcinoma (KIRC) cohort from TCGA (532 cases). (DOCX 17 kb)

## Abbreviations

5-Aza-dC: 5-Aza-2′-deoxycytidine
BGS: Bisulfite genomic sequencing PCR
ccRCC: Clear cell renal cell carcinoma
chRCC: Chromophobe renal cell carcinoma
E-cadherin, CDH1: Epithelial cadherin
EdU: Ethynyl-2-deoxyuridine
EMT: Epithelial-mesenchymal transition
IHC: Immunohistochemistry
ISUP: International Society of Urological Pathology
MSP: Methylation-specific PCR
N-cadherin, CDH2: Neural cadherin
PCR: Polymerase Chain Reaction
pRCC: Papillary renal cell carcinoma
qRT-PCR: Quantitative real-time PCR
RCC: Renal cell carcinoma
RNA-Seq: High-throughput cDNA sequencing
RT-PCR: Reverse transcription PCR
T: Trichostatin A
TCGA: The Cancer Genome Atlas
TJs: Tight junctions
