# Supplementary material for: Downregulation of CLDN7 due to promoter hypermethylation is associated with human clear cell renal cell carcinoma progression and poor prognosis
Source: J Exp Clin Cancer Res. 2018 Nov 14;37:276. doi: 10.1186/s13046-018-0924-y (PMC6234584; doi:10.1186/s13046-018-0924-y)
Supplement: Supplementary file 1 — Table S1. The clinicopathological features of 120 ccRCC patients from Peking University First Hospital. (DOCX 14 kb) [file 13046_2018_924_MOESM1_ESM.docx]

| Application & Date | Characteristics | Number of cases |
| --- | --- | --- |
| For RNA extraction (n=12) | Median age and age range (years) | 60 (49-76) |
| Collecting from October 2017 to December 2017 | Gender |  |
|  | Male/ Female | 7/5 |
|  | Stage |  |
|  | I/II/III/IV | 8/1/2/1 |
|  | Grade |  |
|  | G1/G2/G3 | 3/6/3 |
| For DNA extraction (n=108) | Median age and age range (years) | 56 (21-86) |
| Collecting from April 2012 to September 2012 | Gender |  |
|  | Male/ Female | 73/35 |
|  | Stage |  |
|  | I/II/III/IV | 79/7/21/1 |
|  | Grade |  |
|  | G1/G2/G3 | 38/59/11 |
| Stage and Grade, 2010 American Joint Committee on Cancer staging system. | | |

**Table S1. The clinicopathological features of 120 ccRCC patients from Peking University First Hospital.**
