# Supplementary figures and images for: Downregulation of CLDN7 due to promoter hypermethylation is associated with human clear cell renal cell carcinoma progression and poor prognosis
Source: J Exp Clin Cancer Res. 2018 Nov 14;37:276. doi: 10.1186/s13046-018-0924-y (PMC6234584; doi:10.1186/s13046-018-0924-y)

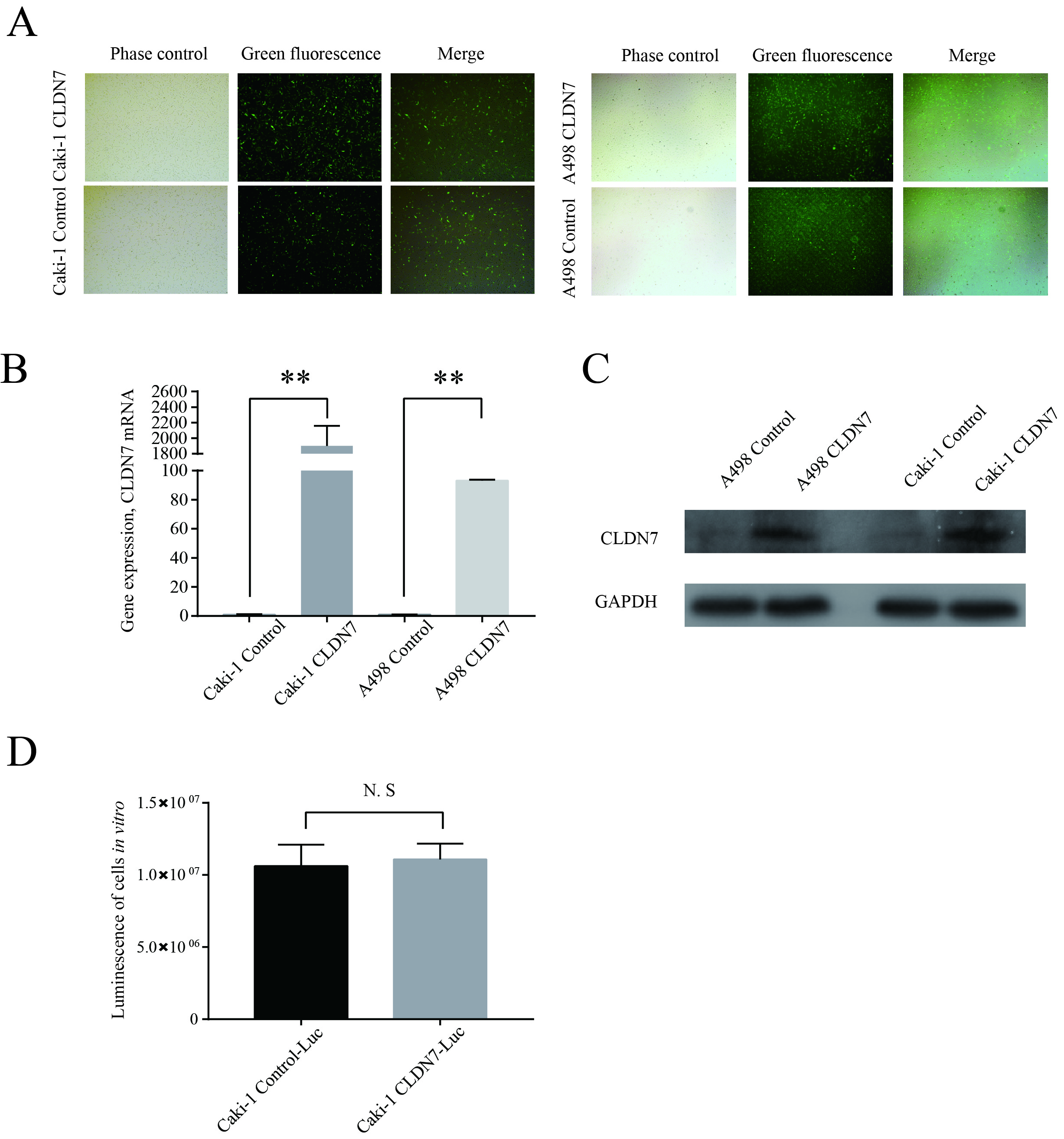

Supplement: Supplementary file 2 — Figure S1. Transfection validation. (JPG 2066 kb) [file 13046_2018_924_MOESM2_ESM.jpg]

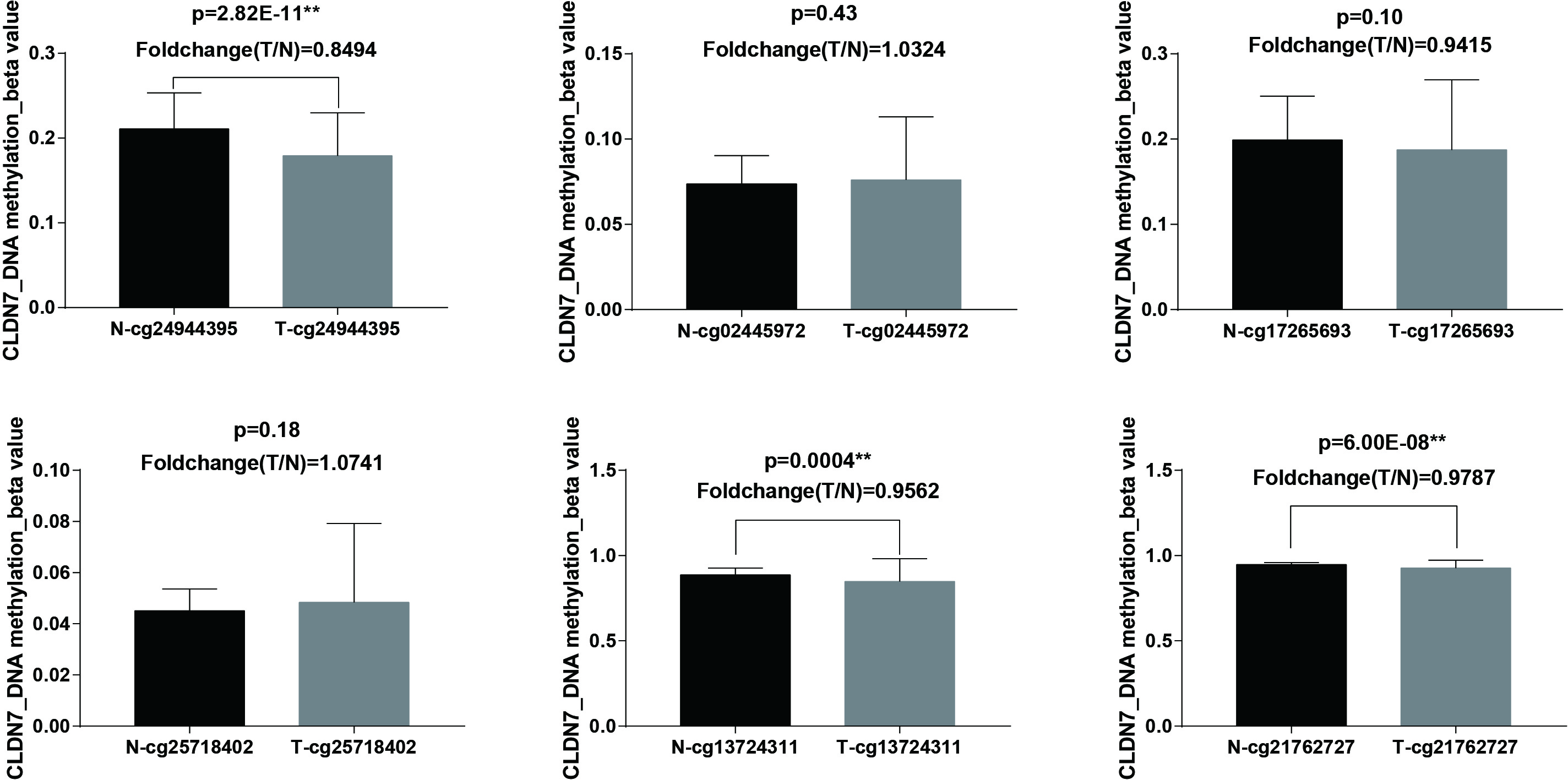

Supplement: Supplementary file 5 — Figure S2. Six DNA methylation sites of CLDN7 promoter that not significantly hypermethylated in TCGA ccRCC dataset. (JPG 1171 kb) [file 13046_2018_924_MOESM5_ESM.jpg]

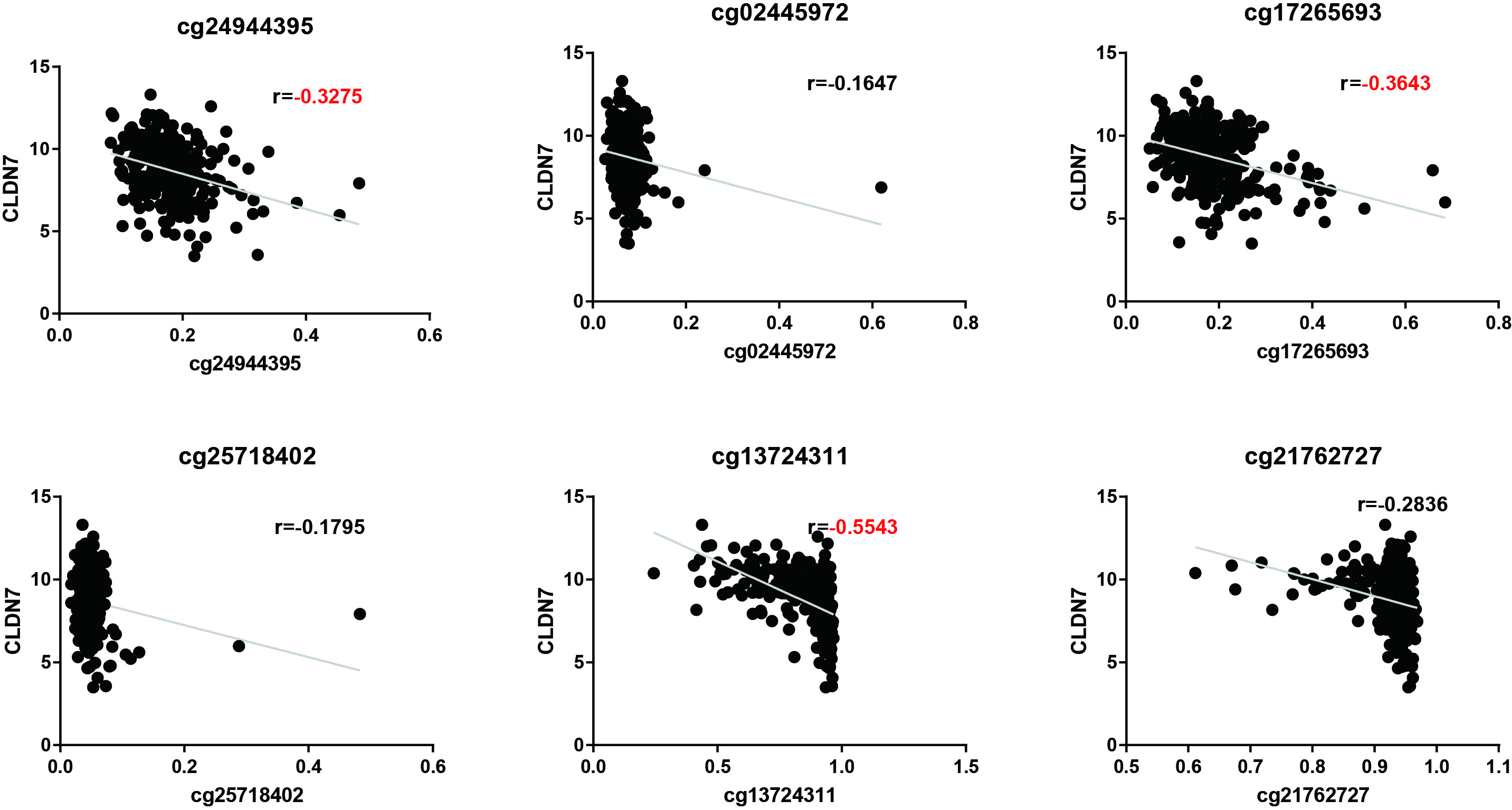

Supplement: Supplementary file 6 — Figure S3. The six DNA methylation regions of CLDN7 promoter that are negatively correlated with CLDN7 mRNA expression in TCGA ccRCC dataset. (JPG 1164 kb) [file 13046_2018_924_MOESM6_ESM.jpg]

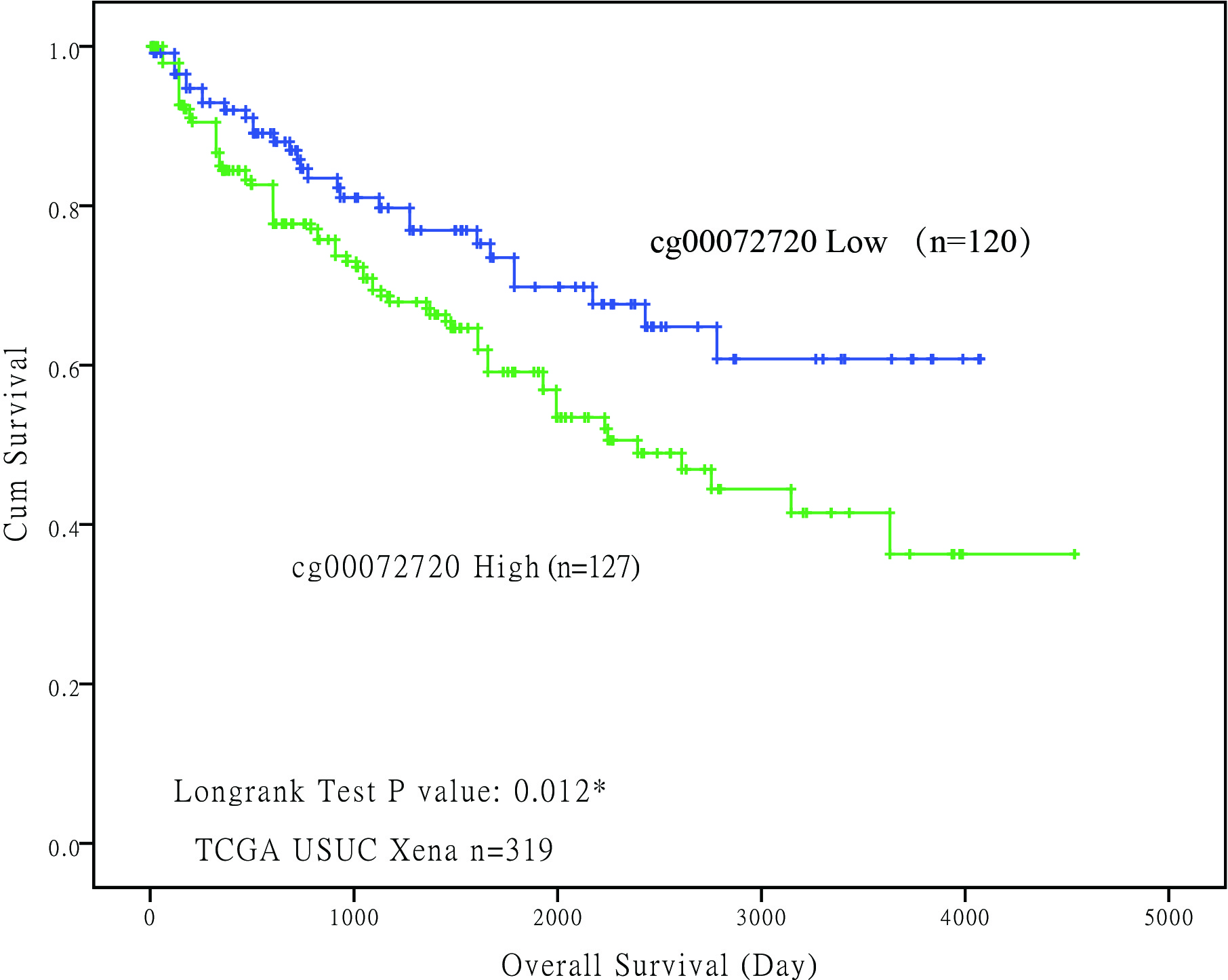

Supplement: Supplementary file 8 — Figure S4. The CLDN7 promoter DNA methylation site, cg00072720, was associated with poor overall survival time while in hypermethylated status. (JPG 470 kb) [file 13046_2018_924_MOESM8_ESM.jpg]

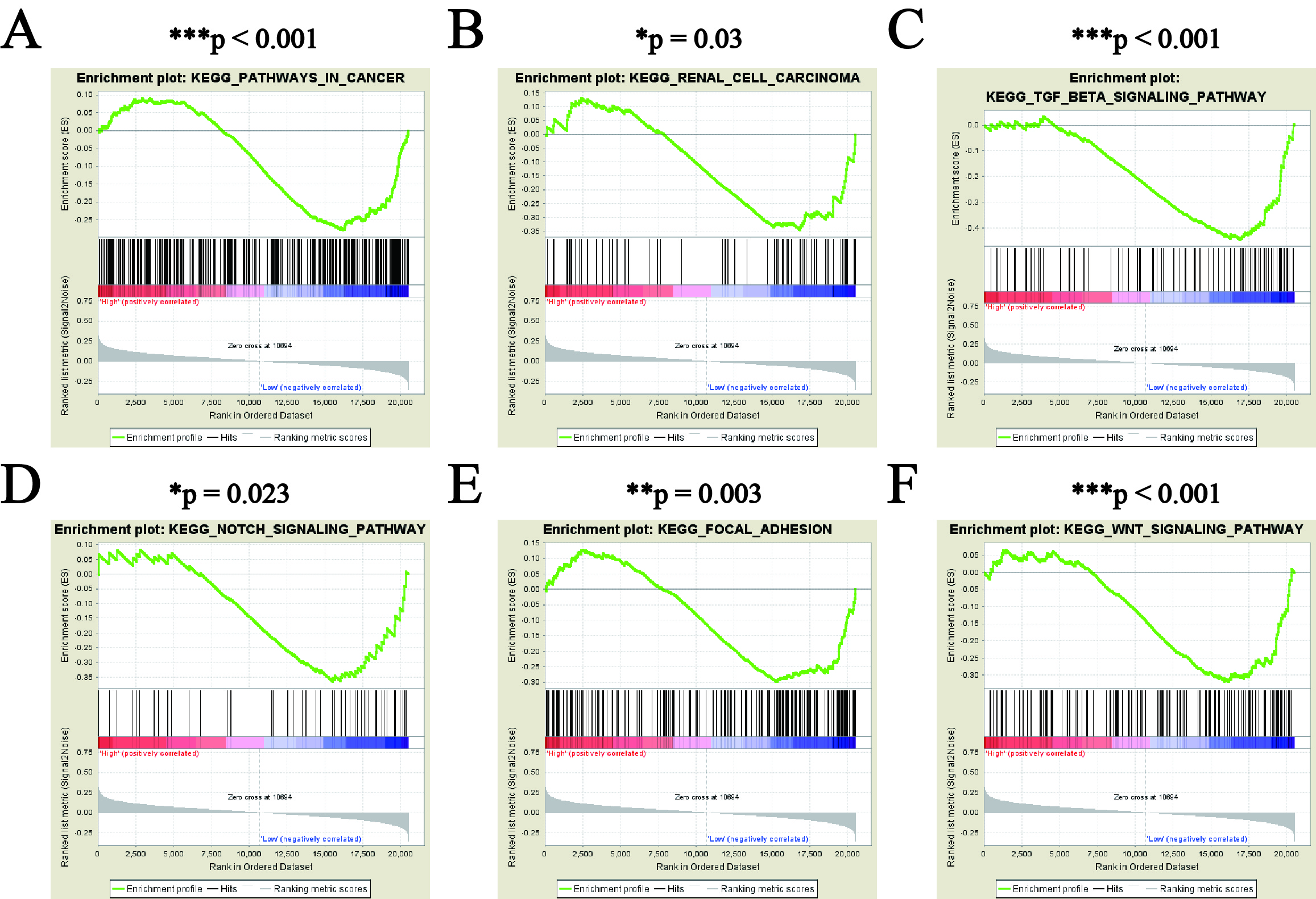

Supplement: Supplementary file 9 — Figure S5. Gene-set enrichment analysis is used to identify the pathways in two different CLDN7 mRNA level groups. (JPG 2095 kb) [file 13046_2018_924_MOESM9_ESM.jpg]
