# Supplementary material for: Downregulation of CLDN7 due to promoter hypermethylation is associated with human clear cell renal cell carcinoma progression and poor prognosis
Source: J Exp Clin Cancer Res. 2018 Nov 14;37:276. doi: 10.1186/s13046-018-0924-y (PMC6234584; doi:10.1186/s13046-018-0924-y)
Supplement: Supplementary file 3 — Table S2. The primers designed for this study. (DOCX 14 kb) [file 13046_2018_924_MOESM3_ESM.docx]

| Gene | Application |  | Sequence | Product |
| --- | --- | --- | --- | --- |
| CLDN7 | qRT-PCR | F | 5’-TTCATCGTGGCAGGTCTT-3’ | 181bp |
|  |  | R | 5’-AGGAACAGGAGAGCAGTG-3’ |  |
| GAPDH | qRT-PCR | F | 5’ -GACAACAGCCTCAAGATCATCAG-3’ | 104bp |
|  |  | R | 5’ -ATGAGTCCTTCCACGATACCA-3’ |  |
| CLDN7 | RT-PCR | F | 5’-CCACTCGAGCCCTAATGGTG-3’ | 266bp |
|  |  | R | 5’-GGTACCCAGCCTTGCTCTCA-3’ |  |
| GAPDH | RT-PCR | F | 5’-ACCACAGTCCATGCCATCAC-3’ | 452bp |
|  |  | R | 5’-TCCACCACCCTGTTGCTGTA-3’ |  |
| CLDN7 | MSP | F | 5’-GACGTTAGGTTATTTTCGGTC-3’ | 259bp |
|  |  | R | 5’-AAACGCGTTTCTAAACGCCG-3’ |  |
| CLDN7 | USP | F | 5’-TGGGGAAAGGGTGGTGTTG-3’ | 182bp |
|  |  | R | 5’-TTACCCAATTTTAACCACCAC-3’ |  |
| CLDN7 | GSB | F | 5’-TTTTTTTATTTGGGTAAGGAGG-3’ | 258bp |
|  |  | R | 5’-GGATTTATAGTTTAGGATATTTTGG-3’ |  |
| F, forward. R, reverse | | | | |

**Table S2. The primers designed for this study.**
