# Supplementary material for: Downregulation of CLDN7 due to promoter hypermethylation is associated with human clear cell renal cell carcinoma progression and poor prognosis
Source: J Exp Clin Cancer Res. 2018 Nov 14;37:276. doi: 10.1186/s13046-018-0924-y (PMC6234584; doi:10.1186/s13046-018-0924-y)
Supplement: Supplementary file 4 — Table S3. Correlation between CLDN7 expression and 653 clinicopathological features in 534 ccRCC patients from TCGA. (DOCX 15 kb) [file 13046_2018_924_MOESM4_ESM.docx]

**Table S3. Correlation between CLDN7 expression and clinicopathological features in 534 ccRCC patients from TCGA (*, p<0.05. **, p<0.01).**

| Clinicopathological features | | CLDN7 mRNA | | P value |
| --- | --- | --- | --- | --- |
|  |  | Low | High |  |
| Additional radiation therapy | No | 96 | 6 | 0.284 |
|  | Yes | 41 | 5 |  |
| Age(median, year) | <=61 | 247 | 36 | 0.191 |
|  | >61 | 228 | 23 |  |
| Gender | Female | 175 | 13 | 0.025* |
|  | Male | 300 | 46 |  |
| Hemoglobin | Normal | 166 | 20 | 0.711 |
|  | Low | 232 | 30 |  |
|  | Elevated | 5 | 0 |  |
| Laterality | Bilateral | 1 | 0 | 0.897 |
|  | Left | 223 | 29 |  |
|  | Right | 251 | 30 |  |
| Histologic grade | G1 | 12 | 2 | 0.251 |
|  | G2 | 198 | 31 |  |
|  | G3 | 190 | 17 |  |
|  | G4 | 70 | 6 |  |
| Pathologic M | M0 | 370 | 52 | 0.026* |
|  | M1 | 76 | 3 |  |
| Pathologic N | N0 | 216 | 24 | 0.625 |
|  | N1 | 15 | 1 |  |
| Pathologic T | T1 | 236 | 38 | 0.004** |
|  | T2 | 57 | 12 |  |
|  | T3 | 171 | 9 |  |
|  | T4 | 11 | 0 |  |
| Pathologic stage | Stage I | 230 | 38 | 0.002** |
|  | Stage II | 46 | 11 |  |
|  | Stage III | 116 | 7 |  |
|  | Stage IV | 81 | 3 |  |
| OS | Alive | 309 | 48 | 0.013* |
|  | Dead | 164 | 11 |  |
| DFS | No | 105 | 16 | 0.924 |
|  | Recurrent | 21 | 3 |  |
