# Supplementary material for: Downregulation of CLDN7 due to promoter hypermethylation is associated with human clear cell renal cell carcinoma progression and poor prognosis
Source: J Exp Clin Cancer Res. 2018 Nov 14;37:276. doi: 10.1186/s13046-018-0924-y (PMC6234584; doi:10.1186/s13046-018-0924-y)
Supplement: Supplementary file 7 — Table S4. Correlation between CLDN7 promoter DNA methylation site (cg00072720) and clinicopathological features in 319 ccRCC patients from TCGA. (DOCX 15 kb) [file 13046_2018_924_MOESM7_ESM.docx]

**Table S4. Correlation between CLDN7 promoter DNA methylation site (cg00072720) and clinicopathological features in 319 ccRCC patients from TCGA (*, p<0.05. **, p<0.01).**

| Clinicopathological features | | CLDN7 cg00072720  methylation region | | p value |
| --- | --- | --- | --- | --- |
|  |  | Low | High |  |
| Additional radiation therapy | No | 18 | 38 | 0.911 |
|  | Yes | 10 | 20 |  |
| Age(median, year) | <=61 | 69 | 96 | 0.109 |
|  | >61 | 51 | 103 |  |
| Gender | Female | 44 | 70 | 0.788 |
|  | Male | 76 | 129 |  |
| Hemoglobin | Normal | 50 | 61 | 0.057 |
|  | Low | 50 | 106 |  |
|  | Elevated | 3 | 2 |  |
| Laterality | Bilateral | 0 | 1 | 0.239 |
|  | Left | 49 | 98 |  |
|  | Right | 71 | 100 |  |
| Histologic grade | G1 | 5 | 4 | 0.025* |
|  | G2 | 59 | 74 |  |
|  | G3 | 43 | 80 |  |
|  | G4 | 11 | 39 |  |
| Pathologic M | M0 | 94 | 140 | 0.108 |
|  | M1 | 15 | 38 |  |
| Pathologic N | N0 | 48 | 85 | 0.035* |
|  | N1 | 0 | 8 |  |
| Pathologic T | T1 | 70 | 89 | 0.071 |
|  | T2 | 16 | 25 |  |
|  | T3 | 32 | 79 |  |
|  | T4 | 2 | 6 |  |
| Pathologic stage | Stage I | 69 | 86 | 0.053 |
|  | Stage II | 12 | 19 |  |
|  | Stage III | 22 | 51 |  |
|  | Stage IV | 16 | 43 |  |
| OS | Alive | 91 | 121 | 0.008** |
|  | Dead | 29 | 76 |  |
| DFS | Not | 44 | 58 | 0.763 |
|  | Recurrent | 8 | 9 |  |
