# Supplementary material for: Downregulation of CLDN7 due to promoter hypermethylation is associated with human clear cell renal cell carcinoma progression and poor prognosis
Source: J Exp Clin Cancer Res. 2018 Nov 14;37:276. doi: 10.1186/s13046-018-0924-y (PMC6234584; doi:10.1186/s13046-018-0924-y)
Supplement: Supplementary file 10 — Table S5. Gene-set enrichment analysis between high- and low- CLDN7 group in Kidney clear cell carcinoma (KIRC) cohort from TCGA (532 cases). (DOCX 17 kb) [file 13046_2018_924_MOESM10_ESM.docx]

**Table S5. Gene-set enrichment analysis between high- and low- CLDN7 group in Kidney clear cell carcinoma (KIRC) cohort from TCGA (532 cases).**

| **Down-regulated pathways in high-CLDN7 group** | **NOM p value** |
| --- | --- |
| [KEGG_TGF_BETA_SIGNALING_PATHWAY](http://www.broadinstitute.org/gsea/msigdb/cards/KEGG_TGF_BETA_SIGNALING_PATHWAY) | < 0.0001 |
| [KEGG_BASAL_TRANSCRIPTION_FACTORS](http://www.broadinstitute.org/gsea/msigdb/cards/KEGG_BASAL_TRANSCRIPTION_FACTORS) | 0.002 |
| [KEGG_COLORECTAL_CANCER](http://www.broadinstitute.org/gsea/msigdb/cards/KEGG_COLORECTAL_CANCER) | 0.002 |
| [KEGG_SMALL_CELL_LUNG_CANCER](http://www.broadinstitute.org/gsea/msigdb/cards/KEGG_SMALL_CELL_LUNG_CANCER) | 0.003 |
| [KEGG_AXON_GUIDANCE](http://www.broadinstitute.org/gsea/msigdb/cards/KEGG_AXON_GUIDANCE) | < 0.0001 |
| [KEGG_GLIOMA](http://www.broadinstitute.org/gsea/msigdb/cards/KEGG_GLIOMA) | < 0.0001 |
| [KEGG_DORSO_VENTRAL_AXIS_FORMATION](http://www.broadinstitute.org/gsea/msigdb/cards/KEGG_DORSO_VENTRAL_AXIS_FORMATION) | 0.038 |
| [KEGG_SPLICEOSOME](http://www.broadinstitute.org/gsea/msigdb/cards/KEGG_SPLICEOSOME) | 0.003 |
| [KEGG_WNT_SIGNALING_PATHWAY](http://www.broadinstitute.org/gsea/msigdb/cards/KEGG_WNT_SIGNALING_PATHWAY) | < 0.0001 |
| [KEGG_NEUROTROPHIN_SIGNALING_PATHWAY](http://www.broadinstitute.org/gsea/msigdb/cards/KEGG_NEUROTROPHIN_SIGNALING_PATHWAY) | 0.005 |
| [KEGG_FOCAL_ADHESION](http://www.broadinstitute.org/gsea/msigdb/cards/KEGG_FOCAL_ADHESION) | 0.003 |
| [KEGG_PATHWAYS_IN_CANCER](http://www.broadinstitute.org/gsea/msigdb/cards/KEGG_PATHWAYS_IN_CANCER) | < 0.0001 |
| [KEGG_RENAL_CELL_CARCINOMA](http://www.broadinstitute.org/gsea/msigdb/cards/KEGG_RENAL_CELL_CARCINOMA) | 0.03 |
| [KEGG_CHRONIC_MYELOID_LEUKEMIA](http://www.broadinstitute.org/gsea/msigdb/cards/KEGG_CHRONIC_MYELOID_LEUKEMIA) | 0.018 |
| [KEGG_ACUTE_MYELOID_LEUKEMIA](http://www.broadinstitute.org/gsea/msigdb/cards/KEGG_ACUTE_MYELOID_LEUKEMIA) | 0.028 |
| [KEGG_PANCREATIC_CANCER](http://www.broadinstitute.org/gsea/msigdb/cards/KEGG_PANCREATIC_CANCER) | 0.031 |
| [KEGG_NOTCH_SIGNALING_PATHWAY](http://www.broadinstitute.org/gsea/msigdb/cards/KEGG_NOTCH_SIGNALING_PATHWAY) | 0.023 |
| [KEGG_ENDOMETRIAL_CANCER](http://www.broadinstitute.org/gsea/msigdb/cards/KEGG_ENDOMETRIAL_CANCER) | 0.029 |
| [KEGG_PROSTATE_CANCER](http://www.broadinstitute.org/gsea/msigdb/cards/KEGG_PROSTATE_CANCER) | 0.025 |
| [KEGG_CELL_CYCLE](http://www.broadinstitute.org/gsea/msigdb/cards/KEGG_CELL_CYCLE) | 0.015 |
|  |  |
| **Up-regulated pathways n high-CLDN7 group** | **NOM p value** |
| [KEGG_OXIDATIVE_PHOSPHORYLATION](http://www.broadinstitute.org/gsea/msigdb/cards/KEGG_OXIDATIVE_PHOSPHORYLATION) | < 0.0001 |
| [KEGG_PARKINSONS_DISEASE](http://www.broadinstitute.org/gsea/msigdb/cards/KEGG_PARKINSONS_DISEASE) | < 0.0001 |
| [KEGG_RIBOSOME](http://www.broadinstitute.org/gsea/msigdb/cards/KEGG_RIBOSOME) | < 0.0001 |
| [KEGG_HUNTINGTONS_DISEASE](http://www.broadinstitute.org/gsea/msigdb/cards/KEGG_HUNTINGTONS_DISEASE) | < 0.0001 |
| [KEGG_ALZHEIMERS_DISEASE](http://www.broadinstitute.org/gsea/msigdb/cards/KEGG_ALZHEIMERS_DISEASE) | < 0.0001 |
| [KEGG_LYSOSOME](http://www.broadinstitute.org/gsea/msigdb/cards/KEGG_LYSOSOME) | < 0.0001 |
| [KEGG_ARACHIDONIC_ACID_METABOLISM](http://www.broadinstitute.org/gsea/msigdb/cards/KEGG_ARACHIDONIC_ACID_METABOLISM) | < 0.0001 |
| [KEGG_CITRATE_CYCLE_TCA_CYCLE](http://www.broadinstitute.org/gsea/msigdb/cards/KEGG_CITRATE_CYCLE_TCA_CYCLE) | < 0.0001 |
| [KEGG_GLUTATHIONE_METABOLISM](http://www.broadinstitute.org/gsea/msigdb/cards/KEGG_GLUTATHIONE_METABOLISM) | < 0.0001 |
| [KEGG_ALPHA_LINOLENIC_ACID_METABOLISM](http://www.broadinstitute.org/gsea/msigdb/cards/KEGG_ALPHA_LINOLENIC_ACID_METABOLISM) | 0.004 |
| [KEGG_CARDIAC_MUSCLE_CONTRACTION](http://www.broadinstitute.org/gsea/msigdb/cards/KEGG_CARDIAC_MUSCLE_CONTRACTION) | < 0.0001 |
| [KEGG_PROTEASOME](http://www.broadinstitute.org/gsea/msigdb/cards/KEGG_PROTEASOME) | 0.005 |
| [KEGG_STEROID_BIOSYNTHESIS](http://www.broadinstitute.org/gsea/msigdb/cards/KEGG_STEROID_BIOSYNTHESIS) | 0.004 |
| [KEGG_GLYCOLYSIS_GLUCONEOGENESIS](http://www.broadinstitute.org/gsea/msigdb/cards/KEGG_GLYCOLYSIS_GLUCONEOGENESIS) | 0.003 |
| [KEGG_LINOLEIC_ACID_METABOLISM](http://www.broadinstitute.org/gsea/msigdb/cards/KEGG_LINOLEIC_ACID_METABOLISM) | 0.002 |
| [KEGG_PEROXISOME](http://www.broadinstitute.org/gsea/msigdb/cards/KEGG_PEROXISOME) | < 0.0001 |
| [KEGG_DRUG_METABOLISM_OTHER_ENZYMES](http://www.broadinstitute.org/gsea/msigdb/cards/KEGG_DRUG_METABOLISM_OTHER_ENZYMES) | < 0.0001 |
| [KEGG_METABOLISM_OF_XENOBIOTICS_BY_CYTOCHROME_P450](http://www.broadinstitute.org/gsea/msigdb/cards/KEGG_METABOLISM_OF_XENOBIOTICS_BY_CYTOCHROME_P450) | < 0.0001 |
| [KEGG_VIBRIO_CHOLERAE_INFECTION](http://www.broadinstitute.org/gsea/msigdb/cards/KEGG_VIBRIO_CHOLERAE_INFECTION) | < 0.0001 |
| KEGG_TYROSINE_METABOLISM | 0.007 |
